# Appraisal of clinical practice guidelines and consensus statements on aspirin in pregnancy: a systematic review using the AGREE II instrument

**DOI:** 10.3389/fphar.2026.1924311

**Published:** 2026-10-30

**Authors:** Yajiao Hu, Dongxu Chen, Juan Ni, Xulin Zhang, Sainan Duan, Chao Yu

**Affiliations:** 1 Department of Anesthesiology, West China Second University Hospital, Sichuan University, Chengdu, China; 2 Key Laboratory of Birth Defects and Related Diseases of Women and Children, Ministry of Education, Chengdu, China; 3 Department of Anesthesiology, Sichuan Academy of Medical Sciences & Sichuan Provincial People’s Hospital, University of Electronic Science and Technology of China, Chengdu, China

**Keywords:** AGREE II, appraisal, aspirin, pregnancy, systematic review

## Abstract

**Objective:**

Recommendations regarding the use of aspirin in obstetrics vary across clinical practice guidelines and consensus statements of differing quality. This study aimed to systematically evaluate the quality of these documents, with particular focus on the recommended daily dosage, timing of initiation, and cessation of aspirin in the obstetric setting. This study employed a systematic review and quality appraisal approach using the Appraisal of Guidelines for Research and Evaluation (AGREE) II methodology.

**Methods:**

The study included the most recent versions of all international and national/regional clinical practice guidelines and consensus statements on aspirin use in pregnancy, published in English or Chinese. Data were retrieved from PubMed (11 April 2025), three Chinese academic databases (the Chinese Medical Association Database, Wanfang Data and China National Knowledge Infrastructure) and four guideline databases (11 April 2025). Two reviewers independently screened the searched items and extracted data. Four reviewers independently scored documents using AGREE II. Recommendations from all documents were tabulated and visualized using a color-coded grid.

**Results:**

A total of 32 documents were include, encompassing six obstetric conditions: Antiphospholipid Syndrome (APS), Fetal Growth Restriction (FGR), advanced maternal age, pregnancy loss, stillbirth and hypertension/pre-eclampsia. The included documents demonstrated strong performance in the domains of scope and purpose (median score: 89.67%, inter-quartile range [IQR] 61.11%–98.61%) and clarity of presentation (median 87.93%, IQR 50%–100%). The editorial independence domain also received relatively high score (median 73.59%, IQR 0%–100%). However, performance was lower in the domain of applicability (median 45.08%, IQR 0%–89.58%). Although aspirin 75–100 mg daily represents the most commonly recommended dosage, a broad range of dosing recommendations exists across most clinical scenarios. However, considerable controversy existed regarding the optimal timing of discontinuation, particularly in cases of hypertensive disorders of pregnancy/pre-eclampsia.

**Conclusion:**

Guidelines and consensus statements, which were generally of relatively high quality, demonstrated that most documents support the use of aspirin for specific obstetric complications. Given these substantial variations in dosage ranges, treatment timing, and combination therapy recommendations, developing standardized, evidence-based protocols is essential to improve the consistency and quality of aspirin therapy in obstetric care.

**Systematic Review Registration:**

https://inplasy.com/inplasy-2025-4-0017, identifier INPLASY202540017.

## Introduction

Aspirin is a well-studied and widely used medication belonging to the non-steroidal anti-inflammatory (NSAID) drug class. As an irreversible cyclooxygenase (COX)-1 inhibitor, it exerts anti-inflammatory and anti-platelet effects by modulating autocrine and paracrine eicosanoid signaling (Mitchell and Kirkby, 2019). Study (Warner et al., 2011) has shown that low-dose aspirin (60–150 mg/d) predominantly inhibits COX-1 activity, downregulates thromboxane A2 (TXA2) expression, and increases the prostaglandin I2 (PGI2)/TXA2 ratio, thereby improving hypercoagulability and placental perfusion. In contrast, high-dose aspirin (>150 mg/d) primarily inhibits COX-2 activity, reduces PGI2 production, and may exacerbate the hypercoagulable state.

The use of aspirin in obstetrics has garnered significant attention due to its potential to reduce adverse pregnancy outcomes, including pre-eclampsia (Ninan et al., 2023), intrauterine growth restriction (McCowan et al., 2018), stillbirth and recurrent pregnancy loss (Youssef et al., 2019). To optimize care for specific pregnancy conditions, numerous clinical guidelines and consensus statements on aspirin use in pregnancy have been developed by healthcare institutions and professional societies (Ninan et al., 2023), addressing daily dosage, time of initiation, and timing of cessation. However, the development of such guidelines may be influenced by the expertise and composition of the development groups, potentially leading to variability in quality. Low-quality guidance documents pose risks to individual patients and the broader community, as they may hinder the implementation of recommendations in clinical practice (Scott et al., 2022). In addition, some documents lack clarity or fail to identify priority domains, potentially leading to clinician misunderstanding and suboptimal prioritization clinical actions (Woolf et al., 1999). Therefore, it is essential to systematically analyze and compare existing guidelines and consensus statements on aspirin use in pregnancy to ensure safe and effective maternal and neonatal care.

In this study, we aimed to evaluate the scope, content, and consistency of current guidance or consensus documents on aspirin using in pregnancy. We assessed the methodological quality of included guidelines and consensus statements using the Appraisal of Guidelines for Research and Evaluation II (AGREE II) instrument and reported domain and overall scores.

## Materials and methods

### Study design and participant

This study was conducted in accordance with the Preferred Reporting Items for Systematic Reviews and Meta Analyses for Protocols (Shamseer et al., 2015). The study protocol was prospectively registered at the International Platform of Registered Systematic Review and Meta-analysis Protocol (Registration No INPLASY202540017).

### Intervention and data collection

#### Literature search and selection criteria

We conducted a systematically search of PubMed from inception to 11 April 2025 using a comprehensive search strategy (Supplementary Table 1) to identify guidelines and consensus statements on aspirin use in pregnancy. Additionally, three academic databases (the Chinese Medical Association Database, Wanfang Data, and China National Knowledge Infrastructure; (Supplementary Table 2) and four guideline databases were searched from inception to 11 April 2025, using search strategies tailored to different databases (Supplementary Table 3). To ensure completeness, we screened the reference lists of pertinent clinical practice guidelines and review articles to identify any additional records.

We included the latest versions of international and national/regional clinical practice guidelines and consensus statements for the use of aspirin in pregnancy published in English or Chinese. Clinical practice guidelines and consensus statements were defined as documents providing recommendations for patient care, derived either from a systematic review of existing evidence or from the collective opinions of an expert panel (Graham et al., 2011). The exclusion criteria were as follows: (1) original investigation, study protocols, comments on existing guidelines or consensus, and conference abstracts or posters; (2) draft documents that were under development or not finalized; and (3) previous documents that had been replaced by updated versions from the same organization.

Two reviewers (CY and YJH) independently screened all records. Full texts for eligible guidelines and consensus statements were retrieved and examined for eligibility. Reasons for exclusion at the full-text stage were documented (Supplementary Table 4). Disagreements were resolved through discussion with a third reviewer (DXC).

#### Data extraction

For each included document, we extracted publication year, funding source, and evidence base, as well as recommendations related to aspirin use in pregnancy. Data extraction was performed independently by two investigator (CY and YJH) and cross-checked by a third investigator (NH). Any discrepancies were resolved by discussion to reach resolution.

### Outcomes measurement

#### Appraisal of guidance documents

Four independent reviewers (CY, YJH, NH, and DXC) appraised the included documents using the AGREE II tool (Brouwers et al., 2010), a validated instrument widely used to assess the quality of clinical practice guidelines and consensus statements. The AGREE II tool consists of 23 items across six domains: scope and purpose, stakeholder involvement, rigor of development, clarity of presentation, applicability, and editorial independence. Each item is scored on a seven-point Likert scale ranging from 1 (strongly disagree) to 7 (strongly agree). Methods for domain scoring are detailed in the Supplementary Material.

Prior to the review, all appraisers completed the official online training tutorial (http://www.center-agree-ii-training-tools/; Accessed 1 September 2023) to ensure standardization. Detailed instruction from the AGREE II User’s Manual (Brouwers et al., 2010) and objective scoring criteria (Supplementary Table 5) were used to promote scoring consistency.

#### Synthesis of recommendations

We systematically extracted recommendations addressing key clinical questions from the included guidance and consensus documents. Recommendations were extracted by two investigators (CY and YJH) and verified by two others (NH and DXC). To visualize concordance and divergence across documents, a color-coded matrix was used to illustrate differences.

### Statistical analysis

For each AGREE II domain, we summarized scores as median and inter-quartile range (IQR). Following prior literature, domains with standardized scores >50% were considered higher-quality guidance (Huang et al., 2024). The most frequently endorsed recommendation for each clinical question was used as the reference. Partially consistent recommendation was defined as one those that overlapped with, but were not identical to, the reference content.

## Results

### Study selection

We identified 5,398 records in total. After title and abstract screening, 5,342 were excluded as ineligible (Figure 1). The remaining 56 records underwent full-text assessment, of which 14 were excluded for not meeting predefined criteria. Ultimately, 32 documents were included (Committee on Practice Bulletins—Obstetrics and American College of Obstetricians and Gynecologists, 2012; Chinese Medical Association, 2020; Vayssière et al., 2015; Fetal Medicine Subgroup et al., 2019; Martins et al., 2020; American College of Obstetricians and Gynecologists, 2021; Kingdom et al., 2023; Morris et al., 2024; American College of Obstetricians and Gynecologists, 2022; Society of Perinatal Medicine et al., 2024; Huchon et al., 2016; Queensland Clinical Guidelines, 2022; Obstetric Group of the Obstetrics and Gynecology Branch of the Chinese Medical Association and Expert Consensus Writing Group on Diagnosis and Treatment of Recurrent Miscarriage, 2022; Regan et al., 2023; Bender Atik et al., 2023; Ladhani et al., 2018; Garabedian et al., 2025; American College of Obstetricians and Gynecologists, 2018; Ministry of Health, 2018; Poon et al., 2019; American College of Obstetricians and Gynecologists, 2020; Group on Hypertensive Disorders in Pregnancy and Obstetrics and Gynecology Branch of the Chinese Medical Association, 2020; Stefanovic, 2023; Queensland Clinical Guidelines, 2021; Henderson et al., 2021; World Health Organization, 2021; Magee et al., 2022; Shanmugalingam et al., 2024; Barry et al., 2023; National Institute for Health and Care Excellence, 2019; Association of Ontario Midwives, 2023; The Royal Australian and New Zealand College of Obstetricians and Gynaecologists, 2024). Detailed of the excluded documents are presented in Supplementary Table 4.

**FIGURE 1:**
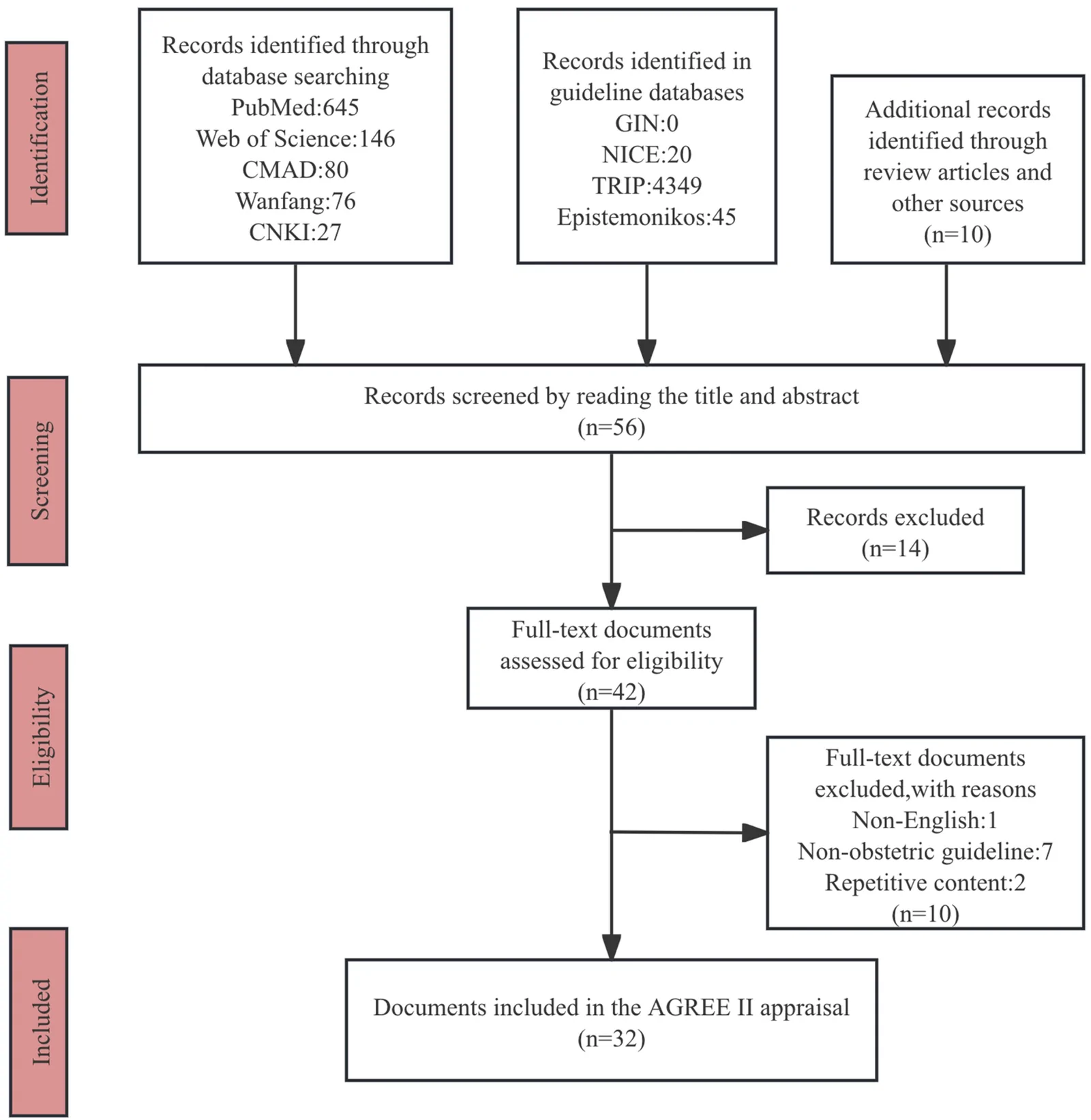
Flow diagram for literature search. AGREE, Appraisal of Guidelines for Research and Evaluation; CNKI, China National Knowledge Infrastructure; CMAD, Chinese Medical Association Database; GIN, Guidelines International Network; NICE, National Institute for Health and Care Excellence; TRIP, Turning Research into Practice Database.

### Characteristics of included guidelines

The 32 included documents on aspirin use in obstetrics, publishing between 2012 and 2024, were categorized into six clinical areas, including antiphospholipid syndrome (APS), fetal growth restriction (FGR), advanced maternal age, pregnancy loss, stillbirth and hypertension/pre-eclampsia (Table 1). Specifically, we identified two on APS (Committee on Practice Bulletins—Obstetrics and American College of Obstetricians and Gynecologists, 2012; Chinese Medical Association, 2020), six on FGR (Vayssière et al., 2015; Fetal Medicine Subgroup et al., 2019; Martins et al., 2020; American College of Obstetricians and Gynecologists, 2021; Kingdom et al., 2023; Morris et al., 2024), two on advanced maternal age (American College of Obstetricians and Gynecologists, 2022; Society of Perinatal Medicine et al., 2024), five on pregnancy loss (Huchon et al., 2016; Queensland Clinical Guidelines, 2022; Obstetric Group of the Obstetrics and Gynecology Branch of the Chinese Medical Association and Expert Consensus Writing Group on Diagnosis and Treatment of Recurrent Miscarriage, 2022; Regan et al., 2023; Bender Atik et al., 2023), two on stillbirth (Ladhani et al., 2018; Garabedian et al., 2025) and 15 on hypertension/pre-eclampsia (American College of Obstetricians and Gynecologists, 2018; Ministry of Health, 2018; Poon et al., 2019; American College of Obstetricians and Gynecologists, 2020; Group on Hypertensive Disorders in Pregnancy and Obstetrics and Gynecology Branch of the Chinese Medical Association, 2020; Stefanovic, 2023; Queensland Clinical Guidelines, 2021; Henderson et al., 2021; World Health Organization, 2021; Magee et al., 2022; Shanmugalingam et al., 2024; Barry et al., 2023; National Institute for Health and Care Excellence, 2019; Association of Ontario Midwives, 2023; The Royal Australian and New Zealand College of Obstetricians and Gynaecologists, 2024). Across categories, most documents focused on daily dose, timing of initiation, and timing of discontinuation; several also addressed co-therapy with other agents and the timing of administration (Figure 2).

**TABLE 1:** Characteristics of included guidelines and consensus statements.

Document	Issuing organization	Country	Funding body	Target population	Target audience	Guideline development group	Guideline review	Guideline update	Evidence base	LOE	SOR
APS											
ACOG-2012 (Committee on Practice Bulletins—Obstetrics and American College of Obstetricians and Gynecologists, 2012)	ACOG	USA	NG	APS	NG	Committee on practice bulletins	NG	Replace 2011	CS	-	+
CMA-2020 (Chinese Medical Association, 2020)	CMA	China	NG	APS	NG	Chin J Perinat Med	NG	NG	CS	-	-
FGR											
FCOG-2015 (Vayssière et al., 2015)	FCOG	France	NG	FGR	NG	FCOG	Peer reviewers	NG	SLR	+	+
CMA-2019 (Fetal Medicine Subgroup et al., 2019)	CMA	China	National key research and development program	FGR	Obstetrician	CMA	External review	NG	CS	+	+
SMFM-2020 (Martins et al., 2020)	SMFM	USA	None	FGR	NG	SMFM	Internal peer review	18-24 months	CS	+	+
ACOG-2021 (American College of Obstetricians and Gynecologists, 2021)	ACOG	USA	NG	FGR	NG	ACOG	NG	2019	NG	+	-
SOGC-2023 (Kingdom et al., 2023)	SOGC	Canada	NG	All pregnant patients with a singleton pregnancy	Obstetricians, family physicians, nurses, midwives, maternal-fetal medicine specialists, radiologists, and other	SOGC	NG	5 years	SLR	+	+
RCOG-2024 (Morris et al., 2024)	RCOG	England	None	FGR	Health care professionals	RCOG	peer review	2002-2013	SLR	+	+
Advanced maternal age											
ACOG-2022 (American College of Obstetricians and Gynecologists, 2022)	ACOG	USA	​	None	Clinicians	ACOG	NG	NG	CS	+	+
CMA-2024Society of Perinatal Medicine et al., 2024)	CMA	China	NG	Advanced maternal age	Clinicians	Society of Perinatal Medicine	Peer review	NG	CS	+	+
Pregnancy loss											
FRENCH-2016 (Huchon et al., 2016)	FCOG	France	NG	Pregnancy loss	Gynecologists, obstetricians, midwives and general practitioners.	FCOG	External review	NG	CS	+	+
QUEENSLAND-2022 (Queensland Clinical Guidelines, 2022)	Queensland Clinical Guidelines	Australia	Healthcare Improvement Unit, Queensland Health	Pregnant with early pregnant loss	Health professionals	Queensland Clinical Guidelines Steering Committee	NG	NG	CS	-	-
CMA-2022 (Obstetric Group of the Obstetrics and Gynecology Branch of the Chinese Medical Association and Expert Consensus Writing Group on Diagnosis and Treatment of Recurrent Miscarriage, 2022)	CMA	China	NG	Pregnant with recurrent spontaneous	NG	Obstetrics and Gynecology Branch of the Chinese Medical Association	NG	2016	CS	-	-
RCOG-2023 (Regan et al., 2023	RCOG	England	NO	Women with recurrent miscarriage	Gynecological and obstetric	RCOG	NG	1998-2003-2011	CS	+	+
ESHRE-2023 (Bender Atik et al., 2023)	ESHRE	ESHRE	NG	Women with recurrent pregnancy loss	Stakeholder, healthcare professionals	ESHRE	External review	2022	CS	+	+
Stillbirth											
SOGC-2018 (Ladhani et al., 2018)	SOGC	Canada	Tommy’s (Charity)	Women and families presenting for care following a pregnancy affected by stillbirth or other causes perinatal loss	Clinicians involved in the obstetric management of women with a history of stillbirth or other causes of perinatal loss	SOGC	External review	None	SLR and CS	+	+
FCOG-2024 (Garabedian et al., 2025)	FCOG	France	None	General population	Obstetrician-Gynecologists, midwives, general practitioners, anesthesiologists, geneticists, and pathologists	Multi	External review	NG	SLR	+	+
Hypertension or/and Pre-eclampsia											
ACOG-2018 (American College of Obstetricians and Gynecologists, 2018)	ACOG	USA	NG	All pregnant women	NG	ACOG	NG	NG	CS	-	-
NEW ZEALAND-2018 (Ministry of Health, 2018)	Ministry of Health	New Zealand	Ministry of Health	Women at risk of and/or presenting with hypertensive disorders in pregnancy	The providers of maternity care	Multi	The Guideline development team	NG	SLR	+	+
FIGO-2019 (Poon et al., 2019)	FIGO	Multi	PerkinElmer Inc, Waltham, USA	Pre-eclampsia of pregnant women	Healthcare providers; healthcare delivery organizations and providers; professional organizations	FIGO	NG	NG	SLR and CS	+	+
ACOG-2020 (American College of Obstetricians and Gynecologists, 2020)	ACOG	USA	Institutional	Hypertension and Pre-eclampsia of pregnant women	Obstetrician-Gynecologists	ACOG	NG	2018-2020	SLR	+	+
CMA-2020 (Group on Hypertensive Disorders in Pregnancy and Obstetrics and Gynecology Branch of the Chinese Medical Association, 2020)	OG-CMA	China	NG	Hypertension of pregnant women	NG	OG-CMA	NG	2015-2020	SLR and CS	+	+
IAPM-2021 (Stefanovic, 2023)	IAPM	Multi	None	NG	NG	IAPM	NG	NG	NG	_	_
QUEENSLAND-2021 (Queensland Clinical Guidelines, 2021)	Queensland Health	Australia	Healthcare Improvement Unit, Queensland Health	NG	Health professionals in Queensland public and private maternity and neonatal services	Queensland Health	NG	Planned in 2026	NG	_	_
USPSTF-2021 (Henderson et al., 2021)	US Preventive Services Task Force	USA	The Agency for Healthcare Research and Quality	Pre-eclampsia of pregnant women	NG	US Preventive Services Task Force	NG	2014-2021	SLR	+	+
WHO-2021 (World Health Organization, 2021)	WHO	Multi	NG	Pre-eclampsia of pregnant women	Health professionals, midwives, nurses, general medical practitioners, and obstetricians	WHO	External review	None	SLR and CS	+	+
SOGC-2022Magee et al., 2022)	SOGC	Canada	NG	Pregnant women	All health care providers	SOGC	External review	2014-2022	SLR and CS	+	+
SOMANZ-2023 (Shanmugalingam et al., 2024)	SOMANZ	Australia and New Zealand	None	NG	NG	SOMANZ	NG	Planned in 2029	SLR and CS	+	+
USPSTF-2023 (Barry et al., 2023)	US Preventive Services Task Force	USA	The Agency for Healthcare Research and Quality	Hypertension of pregnant women	NG	US Preventive Services Task Force	NG	2017-2023	SLR	+	+
NICE-2023 (National Institute for Health and Care Excellence, 2019)	NICE	UK	NG	NG	NG	NICE	NG	2019-2023	SLR	+	+
AOM-2023 (Association of Ontario Midwives, 2023)	AOM	Canada	The Ministry of Health	Hypertensive disorders of pregnant women	Midwives	AOM	NG	2012-2023	SLR	+	+
RANZCOG-2024 (The Royal Australian and New Zealand College of Obstetricians and Gynaecologists, 2024)	RANZCOG	Australia and New Zealand	RANZCOG	NG	Health professionals and consumers	RANZCOG	External review	2015-2024	SLR	+	+

LOE, level of evidence; SOR, strength of recommendation; ACOG, American College of Obstetricians and Gynecologists; USA, the united states of America; NG, not given; CS, consensus guidelines; APS, antiphospholipid syndrome; CMA, Chinese Medical Association; FCOG, French College of Gynaecologists and Obstetricians; FGR, fetal growth restriction; SLR, systematic literature reviews; SMFM, Society for Maternal-Fetal Medicine; SOGC, Society of Obstetricians and Gynecologists of Canada; RCOG, Royal College of Obstetricians and Gynaecologists; ESHRE, European Society of Human Reproduction and Embryology; FIGO, International Federation of Gynecology and Obstetrics; IAPM, International Academy of Perinatal Medicine; USPSTF, US Preventive Services Task Force; WHO, World Health Organization; SOMANZ, Society of Obstetric Medicine Medicine and New Zealand; NICE, National Institute for Health and Care Excellence; AOM, Association of Ontario Midwives; RANZCOG, Royal Australian and New Zealand College of Obstetricians and Gynaecologists.

**FIGURE 2:**
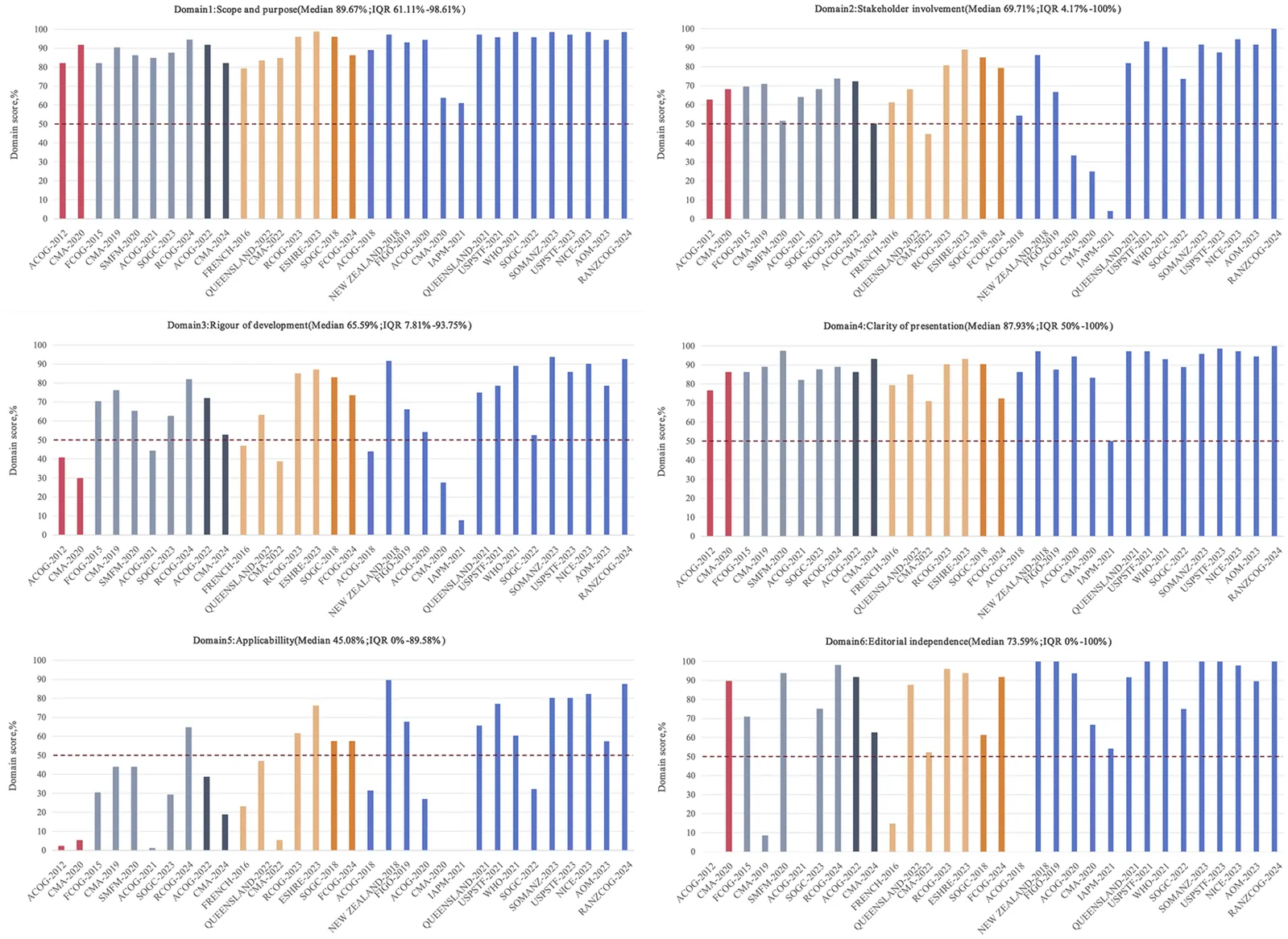
Summary of most recommendations for the use of aspirin in obstetric situation by included guidance document. ACOG, American College of Obstetricians and Gynecologists; CMA, Chinese Medical Association; FCOG, French College of Gynaecologists and Obstetricians; SMFM, Society for Maternal-Fetal Medicine; SOGC, Society of Obstetricians and Gynecologists of Canada; RCOG, Royal College of Obstetricians and Gynaecologists; ESHRE, European Society of Human Reproduction and Embryology; SOGC, Society of Obstetricians and Gynecologists of Canada; FIGO, International Federation of Gynecology and Obstetrics; IAPM, International Academy of Perinatal Medicine; USPSTF, US Preventive Services Task Force; WHO, World Health Organization; SOMANZ, Society of Obstetric Medicine and New Zealand; NICE, National Institute for Health and Care Excellence; AOM, Association of Ontario Midwives; RANZCOG, Royal Australian and New Zealand College of Obstetricians and Gynaecologists; ASA, American Society of Anesthesiologists; LMWH, Low molecular weight heparin; NG, Not Given; NA, Not Applicable.

### Appraisal of guidelines

Four independent reviewers rated each document using AGREE II domain scores. Summary scores are shown in Figure 3 and reported numerically in Online Supplementary Table 5, with item-level mean scores in Online Supplementary Table 6. Overall methodological quality was generally high, with most guidelines scoring 80%–100% in multiple domains, although some exhibited lower performance in specific domains. Among FGR guidelines, the RCOG document published in 2024 had the highest scores (Morris et al., 2024). For advanced maternal age, the 2022 ACOG guidance performed best across most domains (American College of Obstetricians and Gynecologists, 2022). For pregnancy loss, the 2023 ESHRE guideline achieved the highest scores (Bender Atik et al., 2023). Both the 2018 SOGC (Ladhani et al., 2018) and 2024 FCOG (Garabedian et al., 2025) documents demonstrated high quality except applicability domains. For hypertension/pre-eclampsia, most documents were appraised as high quality.

**FIGURE 3:**
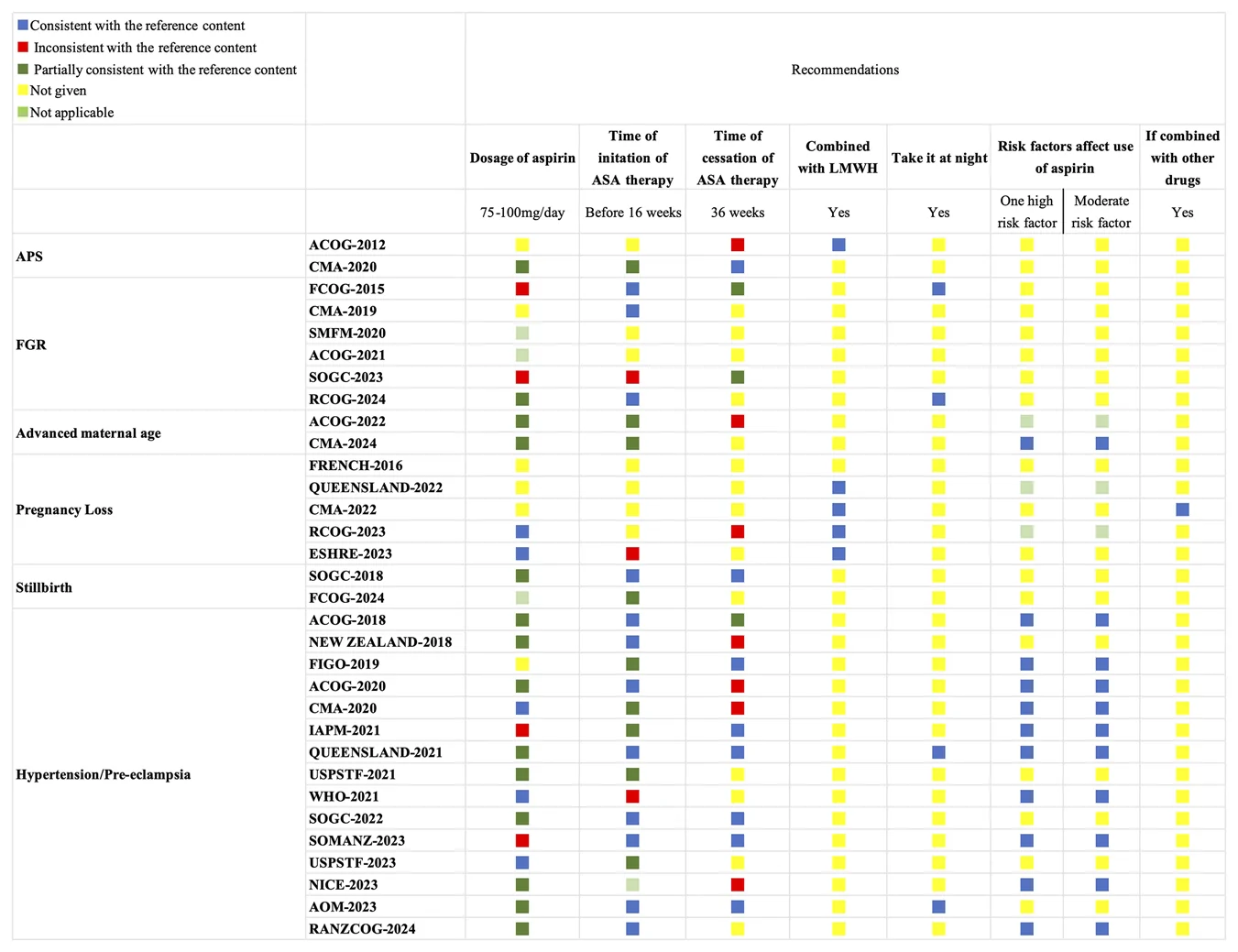
Standardised domain scores for each document. 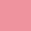 Aspirin in overall pregnancy situation. 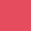 APS 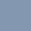 FGR 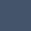 Advanced maternal age 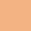 Pregnancy loss 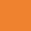 Stillbirth 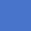 Hypertension/Pre-eclampsia. ACOG, American College of Obstetricians and Gynecologists; CMA, Chinese Medical Association; FCOG, French College of Gynaecologists and Obstetricians; SMFM, Society for Maternal-Fetal Medicine; SOGC, Society of Obstetricians and Gynecologists of Canada; RCOG, Royal College of Obstetricians and Gynaecologists; ESHRE, European Society of Human Reproduction and Embryology; SOGC, Society of Obstetricians and Gynecologists of Canada; FIGO, International Federation of Gynecology and Obstetrics; IAPM, International Academy of Perinatal Medicine; USPSTF, US Preventive Services Task Force; WHO, World Health Organization; SOMANZ, Society of Obstetric Medicine and New Zealand; NICE, National Institute for Health and Care Excellence; AOM, Association of Ontario Midwives; RANZCOG, Royal Australian and New Zealand College of Obstetricians and Gynaecologists; APS, antiphospholipid syndrome; FGR, fetal growth restriction.

By domain (Figure 3), the scope and purpose domain received uniformly high scores (median 89.67%; IQR 61.11%–98.61%), as did clarity of presentation (median 87.93%; IQR 50.00%–100.00%). Editorial independence was also relatively strong (median 73.59%; IQR 0.00%–100.00%). Lower scores were observed for stakeholder involvement (median 69.71%; IQR 4.17%–100.00%) and rigor of development (median 65.59%; IQR 7.81%–93.75%). Applicability had the lowest median score (45.08%; IQR 0.00%–89.58%).

### Synthesis of recommendations

#### The use of aspirin in APS

Two guidelines recommended the use of aspirin for the treatment of APS (Committee on Practice Bulletins—Obstetrics and American College of Obstetricians and Gynecologists, 2012; Chinese Medical Association, 2020). In patients with APS, lower doses of 50–100 mg/day were suggested (Roberge et al., 2017), although precise initiation or discontinuation times were not specified in these guidelines (Table 2). The aspirin dosage depends on the presence of thrombosis (Chinese Medical Association, 2020). The (Chinese Medical Association, 2020) recommended 50–100 mg/day for APS combined with RSA without thrombosis, whereas 100 mg/day was advised for APS combined with RSA and thrombosis.

**TABLE 2:** Recommendations on use of aspirin in guidelines and consensus statements.

Category	Authoring organization (s), year	Inclusion and exclusion criteria for aspirin recommendation	Recommended for aspirin therapy		
			Daily dosage	Initiation	Cessation
APS	ACOG,2012 (Committee on Practice Bulletins—Obstetrics and American College of Obstetricians and Gynecologists, 2012)	Women with APS and a history of stillbirth or recurrent fetal loss but no prior thrombotic history	Low-dose	During pregnancy	6 weeks of postpartum
	CMA,2014 (Chinese Medical Association, 2020)	APS + RSA + no thrombosis	50-100 mg	Entire pregnancy	After 36 weeks
		APS + RSA + thrombosis	100 mg	Entire pregnancy	NG
FGR	FCOG,2015 (Vayssière et al., 2015)	With a history of pre-eclampsia before 34 weeks of gestation, and/or FGR below the fifth percentile with a probable vascular origin (professional consensus)	100-160 mg	16 weeks of gestation	34 weeks of gestation
	CMA,2019 (Fetal Medicine Subgroup et al., 2019)	High-risk factors for pre-eclampsia	Low-dose	16^th^ week of pregnancy	NG
	SMFM,2020 (Martins et al., 2020)	NG	NG	NG	NG
	ACOG,2021 (American College of Obstetricians and Gynecologists, 2021)	NG	NG	NG	NG
	SOCG,2023 (Kingdom et al., 2023)	Women at risk of developing pre-eclampsia	150-162 mg	From the end of the early pregnancy stage	At least 34 weeks of gestation
	RCOG,2024 (Morris et al., 2024)	Women at risk of pre-eclampsia and/or placental dysfunction	100-150 mg	16 weeks ago (11 week to 16^+6^ week)	At 36^+6^ weeks gestation
Advanced maternal age	ACOG,2022 (American College of Obstetricians and Gynecologists, 2022)	Pregnant individuals aged 35 or older in the setting of at least one other moderate risk factor	81 mg	12-16 weeks	Continued daily until delivery
	CMA,2024 (Society of Perinatal Medicine et al., 2024)	One or more moderate to high risk factor for advanced maternal age women	100-150 mg	12-16 weeks	NG
Pregnancy Loss	FRENCH,2016 (Huchon et al., 2016)	Women with both late miscarriage and APS	Low-dose	NG	NG
	Queensland,2022 (Queensland Clinical Guidelines, 2022)	Women with APS	Low-dose	NG	NG
	CMA,2022 (Obstetric Group of the Obstetrics and Gynecology Branch of the Chinese Medical Association and Expert Consensus Writing Group on Diagnosis and Treatment of Recurrent Miscarriage, 2022)	Women with APS	Low-dose	NG	NG
	RCOG,2023 (Regan et al., 2023	Women with APS	75 mg	NG	At least 34 weeks of pregnancy
	ESHRE,2023 (Bender Atik et al., 2023)	Women with APS and a history of three or more pregnancy losses	75-100 mg	Before conception	Until 35 weeks’ gestation or delivery
Still birth	SOGC,2018 (Ladhani et al., 2018)	Women at risk for placental insufficiency	81-162 mg	Before the 16th week of pregnancy	At least 35 weeks, preferably up to 37 weeks or even at delivery
	FCOG,2024 (Garabedian et al., 2025)	Women with APS and a history of fetal death due to a vascular cause	Low-dose	NG	NG
Hypertension/Pre-eclampsia	ACOG,2018 (American College of Obstetricians and Gynecologists, 2018)	More than one of several moderate risk factors for pre-eclampsia	81 mg	Initiated between 12 weeks and 28 weeks of gestation (optimally before 16 weeks)	Continued daily until delivery
	New Zealand,2018 (Ministry of Health, 2018)	Women at high risk of developing pre-eclampsia	100 mg	12-16 weeks	Until immediate delivery
	FIGO,2019 (Poon et al., 2019)	High-risk factors for pre-eclampsia	100-150 mg	11-14^+6^weeks	Until either 36 weeks of gestation, when delivery occurs, or when PE is diagnosed
	ACOG,2020 (American College of Obstetricians and Gynecologists, 2020)	High-risk factors, two or more moderate-risk factors for pre-eclampsia	81 mg	12 weeks, ideally before 16 weeks	28weeks
	CMA,2020 (Group on Hypertensive Disorders in Pregnancy and Obstetrics and Gynecology Branch of the Chinese Medical Association, 2020)	Women at risk of recurrent pre-eclampsia	50-150 mg	12-16 weeks	26-28 weeks
	USPSTF,2021 (Stefanovic, 2023)	Women at high risk for pre-eclampsia	81 mg	After 12 weeks of gestation	NG
	Queensland,2021 (Queensland Clinical Guidelines, 2021)	Moderate to high risk factor or pre-eclampsia	100-150 mg	Before 16 weeks	At 36 weeks’ gestation
	IAPM,2021(Henderson et al., 2021)	With any risk factors for pre-eclampsia	150 mg	From 11 to 14 weeks	36 weeks
​	WHO,2021 (World Health Organization, 2021)	At moderate or high risk of pre-eclampsia	75 mg	12-20 weeks	At 36 weeks’ gestation
	SOGC,2022 (Magee et al., 2022)	Women at increased risk of pre-eclampsia	81 or 162 mg	Before 16 weeks’ gestation	By 36 weeks’ gestation
	SOMANZ,2023 (Shanmugalingam et al., 2024)	Women at high risk of developing pre-eclampsia	150mg	Before 16 weeks	Between 34 weeks’ gestation and birth
	USPSTF,2023 (Barry et al., 2023)	Women at high risk for pre-eclampsia	81 mg	After 12 weeks of gestation	NG
	NICE,2023 (National Institute for Health and Care Excellence, 2019)	High risk/ more than 1 moderate risk factor for pre-eclampsia	75-150 mg	From 12 weeks	The birth of the baby
	AOM,2023 (Association of Ontario Midwives, 2023)	Women at increased risk of developing pre-eclampsia	81-162 mg	Before 16 weeks’ gestation	Between 36 weeks’ gestation and delivery
	RANZCOG,2024 (The Royal Australian and New Zealand College of Obstetricians and Gynaecologists, 2024)	Women with an increased risk of pre-eclampsia	At least 100 mg	Before 16 weeks of gestation	At 36 weeks’ gestation (or earlier if delivery precedes)

ACOG, American College of Obstetricians and Gynecologists; CMA, Chinese Medical Association; RSA, Recurrent Spontaneous Abortion; FCOG, French College of Gynaecologists and Obstetricians; SMFM, Society for Maternal-Fetal Medicine; SOGC, Society of Obstetricians and Gynecologists of Canada; RCOG, Royal College of Obstetricians and Gynaecologists; ESHRE, European Society of Human Reproduction and Embryology; SOGC, Society of Obstetricians and Gynecologists of Canada; FIGO, International Federation of Gynecology and Obstetrics; IAPM, International Academy of Perinatal Medicine; USPSTF, US Preventive Services Task Force; WHO, World Health Organization; SOMANZ, Society of Obstetric Medicine and New Zealand; NICE, National Institute for Health and Care Excellence; AOM, Association of Ontario Midwives; RANZCOG, Royal Australian and New Zealand College of Obstetricians and Gynaecologists; APS, antiphospholipid syndrome; FGR, fetal growth restriction; NG, Not given; PE, Pre-eclampsia.

#### The use of aspirin in FGR

FGR frequently occurs as a consequence of hypertension or pre-eclampsia during pregnancy. Six guidelines recommended the use of aspirin for FGR (Vayssière et al., 2015; Fetal Medicine Subgroup et al., 2019; Martins et al., 2020; American College of Obstetricians and Gynecologists, 2021; Kingdom et al., 2023; Morris et al., 2024). These guidelines were generally of high quality, except for the applicability domain. For FGR management, relatively higher doses of 100–150 mg/day (considerable variability) (Morris et al., 2024) or 150–162 mg/day (Vayssière et al., 2015; Kingdom et al., 2023) were advised (Table 2). Aspirin was typically initiated at 16 weeks of gestation and discontinued at 34 weeks (Vayssière et al., 2015; Fetal Medicine Subgroup et al., 2019; Kingdom et al., 2023; Morris et al., 2024).

In cases of advanced maternal age, a dosage of 81–150 mg/day was recommended, with use initiated between 12 and 16 weeks and continued throughout the pregnancy (Table 2). Martins et al. (2020) Some guidelines suggested that low-molecular-weight heparin (LMWH) was more effective for managing FGR, as highlighted by the Martins et al., (2020) and the (American College of Obstetricians and Gynecologists, 2021).

#### The use of aspirin in advanced maternal age

Two guidelines recommended the use of aspirin for pregnant women of advanced maternal age (Table 2) (American College of Obstetricians and Gynecologists, 2022; Society of Perinatal Medicine et al., 2024). Advanced maternal age was defined as pregnancy at 35 years or older (Ogawa et al., 2017), a population prone to developing pre-eclampsia. These guidelines recommended initiating aspirin between the 12th and 16th weeks of gestation and continuing throughout the pregnancy, with the Chinese Medical Association (2020). (Huchon et al., 2016) suggested 100–150 mg/day, for≥100 mg/day and 150 mg/day may decreased the risk for hypertension in advanced maternal age. The low dose, 81 mg/day was the recommended dosage in American College of Obstetricians and Gynecologists (2020). (American College of Obstetricians and Gynecologists, 2022; Society of Perinatal Medicine et al., 2024).

#### The use of aspirin in pregnancy loss

Five guidelines recommended the use of aspirin for pregnancy loss (Table 2) (Huchon et al., 2016; Queensland Clinical Guidelines, 2022; Obstetric Group of the Obstetrics and Gynecology Branch of the Chinese Medical Association and Expert Consensus Writing Group on Diagnosis and Treatment of Recurrent Miscarriage, 2022; Regan et al., 2023; Bender Atik et al., 2023). Huchon et al., (2016) categorized recurrent pregnancy loss (RPL) into late RPL, recurrent RPL, and unexplained RPL. RPL, often associated with APS, is commonly treated with combination therapy that includes LMWH, which is highly recommended for its efficacy and safety profile. The most commonly recommended dosage of aspirin was 75–150 mg/day for RPL (Huchon et al., 2016; Queensland Clinical Guidelines, 2022; Obstetric Group of the Obstetrics and Gynecology Branch of the Chinese Medical Association and Expert Consensus Writing Group on Diagnosis and Treatment of Recurrent Miscarriage, 2022; Regan et al., 2023). However, several recommendations were provided regarding the timing of initiation and discontinuation for this condition, just Obstetric Group of the Obstetrics and Gynecology Branch of the Chinese Medical Association and Expert Consensus Writing Group on Diagnosis and Treatment of Recurrent Miscarriage, 2022, Regan et al. (2023) defined the discontinuation time was 36 weeks and the (Bender Atik et al., 2023) suggested the beginning time was before conception.

#### The use of aspirin in stillbirth

There are two guidelines recommended the use of aspirin in stillbirth (Table 2) (Ladhani et al., 2018; Garabedian et al., 2025) For stillbirth, dosages of both 81–162 mg/day and 100–150 mg/day (Ladhani et al., 2018). were recommended. Garabedian et al. (2025) specifically recommended low-dose aspirin for preventing stillbirth associated with combined vascular diseases. However, for cases with unknown causes of stillbirth, FCOG does not endorse the preventive use of aspirin (Garabedian et al., 2025). Aspirin treatment was generally initiated at 16 weeks of gestation and continued until 36 weeks or even until delivery (Ladhani et al., 2018; Garabedian et al., 2025).

#### The use of aspirin in hypertension/pre-eclampsia

Aspirin has been shown to play a significant role in preventing and treating pre-eclampsia, a condition characterized by hypertension and proteinuria during pregnancy. Fifteen guidelines recommended the use of aspirin to reduce the risk of pre-eclampsia in high-risk patients (Ministry of Health, 2018; Poon et al., 2019; American College of Obstetricians and Gynecologists, 2020; Group on Hypertensive Disorders in Pregnancy and Obstetrics and Gynecology Branch of the Chinese Medical Association, 2020; Stefanovic, 2023; Queensland Clinical Guidelines, 2021; Henderson et al., 2021; World Health Organization, 2021; Magee et al., 2022; Shanmugalingam et al., 2024; Barry et al., 2023; National Institute for Health and Care Excellence, 2019; Association of Ontario Midwives, 2023; The Royal Australian and New Zealand College of Obstetricians and Gynaecologists, 2024). Risk factors for pregnancy-related hypertension were categorized into high-risk and moderate-risk factors, with aspirin dosages varying accordingly (Poon et al., 2019; American College of Obstetricians and Gynecologists, 2020; Stefanovic, 2023; Queensland Clinical Guidelines, 2021; World Health Organization, 2021; National Institute for Health and Care Excellence, 2019; The Royal Australian and New Zealand College of Obstetricians and Gynaecologists, 2024). Notably, 86% of the guidelines demonstrated high domain scores for quality.

For hypertension and pre-eclampsia, the recommended dosage of aspirin ranged from 75–165 mg/day, often stratified based on the number and severity of risk factors. Initiation time was generally advised at 11 or 12 weeks of gestation, with a preference for starting before 16 weeks. Discontinuation times varied widely, with recommendations ranging from 26–28 weeks, 34 weeks, 36 weeks, or even continuing until delivery. Aspirin should be taken under medical supervision to prevent complications such as thrombosis and to manage symptoms including hypertension and proteinuria (World Health Organization, 2021; Barry et al., 2023). In certain scenarios, co-administration with LMWH or other agents, such as hormonal therapies, was recommended (The Royal Australian and New Zealand College of Obstetricians and Gynaecologists, 2024). However, only a minority of guidelines explicitly suggested nighttime dosing of aspirin (Queensland Clinical Guidelines, 2021; Association of Ontario Midwives, 2023).

## Discussion

This systematic review revealed substantial heterogeneity in aspirin recommendations across clinical practice guidelines and consensus statements for various obstetric conditions, highlighting critical challenges for clinical implementation. These wide dosage windows, coupled with variable timing recommendations and inconsistent guidance on combination therapies, underscore the urgent need for more standardized, evidence-based protocols to optimize aspirin use in obstetric practice and facilitate consistent clinical decision-making across different healthcare settings.

Previous studies has evaluated aspirin use in specific pregnancy complications, such as pre-eclampsia (Ninan et al., 2023). However, these studies just focused on single conditions, whereas this analysis examines aspirin usage across all obstetric conditions, identifying the most frequently recommended applications, including dosage, initiation and cessation timing. While 14 documents address aspirin in hypertension/pre-eclampsia, only 2–3 cover APS, stillbirth and advanced maternal age. Given the increasing prevalence of advanced maternal age pregnancy, APS and stillbirth, future guidelines should prioritize these underrepresented areas.

The evaluation of the documents using the AGREE II tool revealed that these guidelines demonstrate high quality in scope and purpose (domain 1) and clarity of presentation (domain 4), but poor quality in applicability (domain 5). The domains of stakeholder in involvement (domain 2), rigor of development (domain 3) and editorial independence (domain 6) performed relatively better. These AGREE II scores were consistent with previous guideline evaluation studies, which typically showed excellent performance in scope and purpose but poor performance in applicability (Li et al., 2019; Fang et al., 2018). Improving the quality of applicability of such guidelines remains the most challenging aspect, as conducting evaluations and pilot studies requires substantial time and economic resources that may not be available in all regions and countries. Notably, our study found better scores in editorial independence than previous studies, as the majority (73.59%) of documents provided detailed information on funding body influence and conflicts of interest.

Recommended aspirin dosages ranged from 50 mg daily in APS without thrombosis to 165 mg daily in severe pre-eclampsia, reflecting a more than threefold difference that may confound clinical decision-making. The most commonly recommended dose was 75–100 mg daily (Chinese Medical Association, 2020; Regan et al., 2023; Bender Atik et al., 2023; Group on Hypertensive Disorders in Pregnancy and Obstetrics and Gynecology Branch of the Chinese Medical Association, 2020; World Health Organization, 2021; Barry et al., 2023; National Institute for Health and Care Excellence, 2019), endorsed by major organizations including American College of Obstetricians and Gynecologists (2020) and World Health Organization (2021) for pre-eclampsia prevention. National Institute for Health and Care Excellence (2019) adhered to 75 mg daily, citing insufficient evidence for higher doses. However, the study by Lin et al. (2022) found that 100 mg daily aspirin initiated from 12–20 weeks until 34 weeks did not reduce pre-eclampsia incidence in Chinese women with high-risk factors, raising questions about whether conventional low doses are sufficient across different populations and whether inadequate dosing or aspirin resistance may impact efficacy.

Recent evidence suggests that higher doses may be more effective in specific populations. The Magee et al. (2024), Bartho et al. (2025) recommended 150 mg daily in women with prior severe pre-eclampsia or FGR, emphasizing dose-dependent efficacy. Among high-risk obese individuals, 162 mg aspirin compared with 81 mg showed a 78% probability of benefit in decreasing severe pre-eclampsia, with an estimated 12% absolute risk reduction (Amro et al., 2025). Similarly, aspirin 150–162 mg daily starting in the first trimester was associated with lower risk of preterm pre-eclampsia compared to 75–81 mg (Ghesquiere et al., 2023). These findings suggest that patient-specific factors such as body weight, obesity, and disease severity should inform dosing decisions rather than a one-size-fits-all approach. Regional variations in recommendations were also evident. For RPL, Obstetric Group of the Obstetrics and Gynecology Branch of the Chinese Medical Association and Expert Consensus Writing Group on Diagnosis and Treatment of Recurrent Miscarriage (2022), Regan et al. (2023) supported 75 mg daily combined with heparin, while Obstetric Group of the Obstetrics and Gynecology Branch of the Chinese Medical Association and Expert Consensus Writing Group on Diagnosis and Treatment of Recurrent Miscarriage (2022) recommended 100 mg daily as monotherapy. Furthermore, broad dosage ranges within individual guidelines-such as 50–100 mg daily for APS or 100–150 mg daily for advanced maternal age-lacked clear criteria for dose selection, potentially leading to arbitrary clinical decisions.

Beyond dosage inconsistencies, marked discrepancies were observed in the timing of initiation and discontinuation of aspirin therapy. For RPL and FGR, discontinuation at 34 weeks was most commonly recommended (Vayssière et al., 2015; American College of Obstetricians and Gynecologists, 2021; Kingdom et al., 2023; Regan et al., 2023). For stillbirth and APS, 36 weeks was preferred when specified (Chinese Medical Association, 2020; Ladhani et al., 2018). However, for hypertensive disorders and pre-eclampsia, recommendations ranged from 28 weeks (American College of Obstetricians and Gynecologists, 2020), 26–28 weeks (Group on Hypertensive Disorders in Pregnancy and Obstetrics and Gynecology Branch of the Chinese Medical Association, 2020), 36 weeks (Poon et al., 2019; Stefanovic, 2023; Magee et al., 2022), until delivery (Ministry of Health, 2018), 34 weeks to delivery (National Institute for Health and Care Excellence, 2019; Association of Ontario Midwives, 2023), with some guidelines citing bleeding risk minimization as rationale for earlier cessation. The lack of recent research on optimal discontinuation timing, particularly for hypertensive disorders, represents an important gap for future investigation.

Recommendations on combination therapy with LMWH differed substantially. Some guidelines strongly endorsed combined approaches for RPL and FGR (Martins et al., 2020; Huchon et al., 2016; Queensland Clinical Guidelines, 2022; Obstetric Group of the Obstetrics and Gynecology Branch of the Chinese Medical Association and Expert Consensus Writing Group on Diagnosis and Treatment of Recurrent Miscarriage, 2022; Regan et al., 2023; Bender Atik et al., 2023), while others provided limited guidance. Special scenarios, including aspirin use in unexplained stillbirth and nighttime administration, received inconsistent attention despite emerging evidence suggesting potential benefits of chronotherapy (Morris et al., 2024; Garabedian et al., 2025).

The substantial variability identified in this review underscores the need for more precise, population-specific recommendations. Future studies should establish tailored protocols that account for patient characteristics such as body weight, obesity, and disease severity, as research in cardiovascular prevention has shown that aspirin’s effects interact with body size (Rothwell et al., 2018). The lack of large, high-quality randomized controlled trials for many obstetric conditions limits the current evidence base.

Safety considerations must also be addressed. Recent research has raised concerns about increased bleeding risks with aspirin use (Hastie et al., 2021), which must be weighed against potential benefits. While one study found that 162 mg aspirin significantly reduced pre-eclampsia rates without increased bleeding complications (Ayyash et al., 2024), more research across diverse populations is needed. Future guidelines should provide evidence-based recommendations that clearly define treatment timelines, offer explicit guidance on combination therapies, and can be consistently applied across different healthcare settings to enhance the quality of obstetric care.

## Strengths and limitations

Several limitations should be acknowledged. First, this study focused on guideline content and quality assessment rather than evaluating clinical practice impact, which exceeded our scope. Second, inclusion of only English and Chinese language documents may have missed important guidance from other regions, though we sought English versions where available. Third, despite using standardized AGREE II methodology with multiple independent reviewers, the inherent subjectivity of quality assessment introduces potential for unconscious bias and challenges reproducibility across different reviews. These quality assessments should be interpreted as one component of the overall evidence synthesis rather than definitive judgments. Lastly, the delayed updating of older guidelines (e.g., those published in 2012 and 2015) introduces a potential source of evidence bias. As clinical practices and research constantly transform, outdated recommendations risk misaligning with current clinical management. This limitation not only highlights the clinical value of our study but also demonstrates the urgent necessity of standardizing the guideline development process.

## Conclusion

This systematic review reveals a critical paradox in contemporary obstetric practice: despite robust evidence supporting aspirin’s benefits in high-risk pregnancies, substantial inconsistencies in guideline recommendations undermine its optimal application. The marked variability in dosing strategies, treatment timelines, and combination therapy approaches identified across international guidelines reflects not merely regional differences in clinical practice, but fundamental gaps in the evidence base that current consensus-driven approaches cannot adequately address. Particularly concerning is the paucity of guidance for increasingly prevalent conditions such as advanced maternal age and antiphospholipid syndrome, where clinical need is growing but evidence-based protocols remain underdeveloped. Addressing these deficiencies requires moving beyond iterative guideline updates toward a more rigorous framework: one that prioritizes adequately powered, population-specific trials; incorporates patient characteristics into dosing algorithms; and emphasizes implementation science to ensure applicability across diverse healthcare settings.

## Glossary

AGREE II: Appraisal of Guidelines for Research and Evaluation II
APS: Antiphospholipid Syndrome
FGR: Fetal Growth Restriction
NSAID: non-steroidal anti-inflammatory
IQR: inter-quartile range
LOE: level of evidence
SOR: strength of recommendation
ACOG: American College of Obstetricians and Gynecologists
USA: the United States of America
NG: not given
CS: consensus guidelines
CMA: Chinese Medical Association
FCOG: French College of Gynaecologists and Obstetricians
SLR: systematic literature reviews
SMFM: Society for Maternal-Fetal Medicine
SOGC: Society of Obstetricians and Gynecologists of Canada
RCOG: Royal College of Obstetricians and Gynaecologists
ESHRE: European Society of Human Reproduction and Embryology
FIGO: International Federation of Gynecology and Obstetrics
IAPM: International Academy of Perinatal Medicine
USPSTF: US Preventive Services Task Force
WHO: World Health Organization
SOMANZ: Society of Obstetric Medicine and New Zealand
NICE: National Institute for Health and Care Excellence
AOM: Association of Ontario Midwives
RANZCOG: Royal Australian and New Zealand College of Obstetricians and Gynaecologists.
RSA: Recurrent Spontaneous Abortion
LMWH: low-molecular-weight heparin
ASA: American Society of Anesthesiologists
