# Supplementary material for: Appraisal of clinical practice guidelines and consensus statements on aspirin in pregnancy: a systematic review using the AGREE II instrument
Source: Front Pharmacol. 2026 Oct 30;17:1924311. doi: 10.3389/fphar.2026.1924311 (PMC13760202; doi:10.3389/fphar.2026.1924311)
Supplement: Supplementary file 1 [file Table1.docx]

**Supplementary Table 1. Search strategy in PubMed.**

| 1 | aspirin | 77392 |
| --- | --- | --- |
| 2 | ((pregnancy) OR (obstetrics)) AND (aspirin) | 4473 |
| 3 | ((obstetrics) OR (pregnancy)) AND ((guideline) OR (recommendation)) AND (aspirin) | 645 |
| Date of literature search: April 11, 2025 | | |

**Supplementary Table 2. Searches in Chinese databases.**

| **China National Knowledge Infrastructure** | |
| --- | --- |
| [Original search term in Chinese]  (obstetrics) OR (pregnancy) OR (guideline) OR (recommendation) AND (aspirin)  Document type: (guideline) OR (recommendation) | 27 |
| Date of literature search: April 11, 2025 | |
| **Wanfang Data** | |
| [Original search term in Chinese]  ((obstetrics) OR (pregnancy)) AND ((guideline) OR (recommendation)) AND (aspirin) | 76 |
| Date of literature search: April 11, 2025 | |
| **Chinese Medical Association Database** | |
| [Original search term in Chinese]  (obstetrics) OR (pregnancy)  Document type: (guideline) OR (recommendation) | 80 |
| Date of literature search: April 12, 2025 | |

**Supplementary Table 3. Searches in guideline databases.**

| **Databases** | **Date of**  **search** | **Search strategy** | **Results**  **found** | **URL** |
| --- | --- | --- | --- | --- |
| Guidelines  International  Network | 2025/4/11 | ((obstetrics) OR (pregnancy)) AND (aspirin) | 0 | www.g-i-n.net |
| National Institute  for Health and Care  Excellence | 2025/4/11 | ((obstetrics) OR (pregnancy)) AND (aspirin)  filter type: guidance | 20 | www.nice.org.uk |
| Turning Research  Into Practice  Database | 2025/4/11 | ((obstetrics) OR (pregnancy)) AND ((guideline) OR (recommendation)) AND (aspirin) | 4349 | www.tripdatabase.com |
| Epistemonikos  database | 2025/4/11 | ((obstetrics) OR (pregnancy)) AND ((guideline) OR (recommendation)) AND (aspirin) | 45 | www.epistemonikos.org |

**Supplementary Table 4. Excluded studies and reasons for exclusion.**

| **Study** | **Reason for exclusion** |
| --- | --- |
| Application of low dose aspirin in pre-eclampsia | Review |
| Assessment of NICE and USPSTF guidelines for identifying women at high risk of pre-eclampsia for tailoring aspirin prophylaxis in pregnancy: an individual participant data meta-analysis | Meta-analysis |
| Comparison and appraisal of (inter)national recurrent pregnancy loss guidelines | Review |
| Diagnosing and treating antiphospholipid syndrome: a consensus paper | Article |
| Evidence-based national guidelines for the management of suspected fetal growth restriction: comparison, consensus, and controversy | Review |
| Guidelines—similarities and dissimilarities: a systematic review of international clinical practice guidelines for pregnancy hypertension | Review |
| Aspirin guideline publication in individuals with hypertensive disorders and gestational diabetes mellitus of pregnancy | Review |
| Hypertensive Disorders of Pregnancy: A Systematic Review of International Clinical Practice Guidelines | Review |
| Improved implementation of aspirin in pregnancy among dutch gynecologists: surveys in 2016 and 2021 | Article |
| Pregnancy and ischemic stroke: a practical guide to management | Review |
| Prevention of pre-eclampsia with aspirin: a systematic review of guidelines and evaluation of the quality of recommendation evidence | Review |
| Screening for pre-eclampsia risk and prophylaxis with acetylsalicylic acid | Commentary |
| Summary of updated recommendations for primary prevention of cardiovascular disease in women | Summary |
| Expert Consensus on Scientific Prevention and Control of Pre-eclampsia | Non-obstetric guideline |
| Chinese Expert Consensus on Off-label Drug Use for Recurrent Miscarriage with Pre-eclampsia and Antiphospholipid Syndrome | Repetitive content |
| Chinese Expert Consensus on Anti-thrombotic Drug Therapy for Recurrent Miscarriage | Repetitive content |
| La pré-éclampsie: recommendations pour la pratique clinique du collège national des gynécologues obstétriciens français | Non-English |
| Guideline supplement: Hypertension and pregnancy | Supplement |
| A summary of the 2023 Society of Obstetric Medicine of Australia and New Zealand (SOMANZ) hypertension in pregnancy guideline | Summary |
| Diagnosing and treating antiphospholipid syndrome: a consensus paper | Article |
| EULAR recommendations for the management of antiphospholipid syndrome in adults | Non-obstetric guideline |
| Guidelines on the investigation and management of antiphospholipid syndrome | Non-obstetric guideline |
| Impact of the ACOG guideline regarding low-dose aspirin for prevention of superimposed pre-eclampsia in women with chronic hypertension | Non-obstetric guideline |
| Society for maternal-fetal medicine special statement: checklists for pre-eclampsia risk-factor screening to guide recommendations for prophylactic low-dose aspirin | Statement |

Reason for exclusion: Review; Meta-analysis; Article; Commentary; Summary; Non-obstetric guideline; Repetitive content; Non-English; Supplement; Statement.

**Supplementary methods. Calculate the scores.**

The reviewers individually assessed each item using a Likert scale, and the scores were combined to calculate the domain scores. The total score for each domain was calculated by summing the scores of all the items within that domain for each reviewer. This total score was then standardized as a percentage of the maximum possible score for that specific domain. The calculation method is as follows:

The minimum possible score is calculated as: (number of questions) × (number of reviewers) × 1.

The maximum possible score is calculated as: (number of questions) × (number of reviewers) × 7.

Five guidance documents were selected for the pilot scoring phase, and discussions were held to clarify scoring criteria. After completing scoring for all included documents, a meeting was held to discuss items with scores differing by more than one point. Reviewers were then given the opportunity to revise their scores or maintain their original scores, with all original and revised scores, along with reasons for modifying scores, meticulously recorded for quality control purposes.

**Supplementary Table 5. Scores for each individual AGREE II domain items by each reviewer.**

| **Document** | **Domain 1** | | | **Domain 2** | | | **Domain 3** | | | | | | | | **Domain 4** | | | **Domain 5** | | | | **Domain 6** | |
| --- | --- | --- | --- | --- | --- | --- | --- | --- | --- | --- | --- | --- | --- | --- | --- | --- | --- | --- | --- | --- | --- | --- | --- |
| **Item** | **1** | **2** | **3** | **4** | **5** | **6** | **7** | **8** | **9** | **10** | **11** | **12** | **13** | **14** | **15** | **16** | **17** | **18** | **19** | **20** | **21** | **22** | **23** |
| ACOG-2012, APS | | | | | | | | | | | | | | | | | | | | | | | |
| Rev-1 | 7 | 6 | 5 | 5 | 5 | 4 | 7 | 3 | 1 | 4 | 2 | 5 | 0 | 5 | 6 | 4 | 6 | 2 | 0 | 1 | 2 | 1 | 0 |
| Rev-2 | 6 | 6 | 5 | 6 | 4 | 6 | 6 | 4 | 2 | 3 | 3 | 4 | 1 | 6 | 5 | 5 | 7 | 1 | 1 | 1 | 1 | 0 | 0 |
| Rev-3 | 7 | 7 | 5 | 4 | 4 | 4 | 5 | 4 | 1 | 4 | 2 | 5 | 0 | 5 | 5 | 5 | 6 | 0 | 1 | 1 | 2 | 0 | 0 |
| Rev-4 | 7 | 6 | 4 | 5 | 5 | 5 | 7 | 2 | 2 | 2 | 3 | 6 | 1 | 5 | 6 | 5 | 7 | 1 | 1 | 2 | 1 | 0 | 1 |
| CMA-2020, APS | | | | | | | | | | | | | | | | | | | | | | | |
| Rev-1 | 7 | 6 | 5 | 7 | 2 | 4 | 3 | 4 | 1 | 0 | 1 | 5 | 3 | 0 | 6 | 6 | 6 | 1 | 0 | 1 | 2 | 6 | 7 |
| Rev-2 | 7 | 7 | 6 | 6 | 3 | 3 | 4 | 5 | 0 | 1 | 2 | 6 | 4 | 0 | 7 | 7 | 4 | 0 | 0 | 1 | 3 | 6 | 6 |
| Rev-3 | 7 | 6 | 7 | 7 | 6 | 5 | 5 | 5 | 4 | 2 | 5 | 4 | 1 | 2 | 6 | 6 | 6 | 2 | 1 | 0 | 2 | 6 | 7 |
| Rev-4 | 6 | 7 | 7 | 7 | 6 | 5 | 5 | 4 | 3 | 1 | 4 | 3 | 1 | 1 | 7 | 7 | 6 | 2 | 2 | 1 | 3 | 6 | 7 |
| FCOG-2015, FGR | | | | | | | | | | | | | | | | | | | | | | | |
| Rev-1 | 7 | 5 | 4 | 6 | 3 | 4 | 5 | 5 | 7 | 5 | 6 | 5 | 4 | 5 | 7 | 6 | 7 | 2 | 4 | 4 | 3 | 5 | 3 |
| Rev-2 | 6 | 6 | 3 | 5 | 4 | 4 | 5 | 4 | 7 | 6 | 5 | 4 | 5 | 4 | 7 | 7 | 7 | 1 | 5 | 3 | 4 | 4 | 4 |
| Rev-3 | 7 | 6 | 7 | 7 | 5 | 6 | 7 | 6 | 1 | 6 | 6 | 7 | 2 | 7 | 6 | 5 | 6 | 2 | 1 | 2 | 5 | 6 | 7 |
| Rev-4 | 7 | 7 | 6 | 7 | 4 | 7 | 7 | 6 | 1 | 6 | 6 | 7 | 3 | 7 | 6 | 4 | 6 | 1 | 1 | 1 | 6 | 6 | 7 |
| CMA-2019, FGR | | | | | | | | | | | | | | | | | | | | | | | |
| Rev-1 | 7 | 6 | 6 | 5 | 3 | 5 | 6 | 7 | 7 | 7 | 6 | 6 | 5 | 3 | 7 | 7 | 7 | 2 | 4 | 4 | 6 | 3 | 2 |
| Rev-2 | 6 | 7 | 5 | 6 | 4 | 3 | 6 | 7 | 7 | 7 | 7 | 6 | 4 | 2 | 7 | 7 | 7 | 1 | 5 | 4 | 5 | 2 | 3 |
| Rev-3 | 7 | 6 | 7 | 7 | 7 | 5 | 7 | 6 | 7 | 7 | 5 | 6 | 4 | 2 | 6 | 5 | 6 | 6 | 2 | 1 | 4 | 0 | 1 |
| Rev-4 | 7 | 7 | 6 | 7 | 7 | 4 | 7 | 6 | 7 | 7 | 5 | 5 | 3 | 1 | 5 | 6 | 6 | 6 | 1 | 2 | 5 | 0 | 1 |
| SMFM-2020, FGR | | | | | | | | | | | | | | | | | | | | | | | |
| Rev-1 | 7 | 6 | 5 | 4 | 2 | 4 | 2 | 3 | 7 | 3 | 4 | 6 | 5 | 5 | 7 | 7 | 7 | 1 | 5 | 5 | 1 | 5 | 7 |
| Rev-2 | 6 | 6 | 6 | 3 | 1 | 5 | 1 | 4 | 7 | 4 | 3 | 6 | 4 | 5 | 7 | 7 | 7 | 0 | 5 | 4 | 1 | 6 | 7 |
| Rev-3 | 7 | 6 | 6 | 5 | 5 | 5 | 5 | 5 | 5 | 5 | 6 | 7 | 5 | 6 | 7 | 6 | 7 | 5 | 5 | 2 | 6 | 7 | 7 |
| Rev-4 | 6 | 7 | 6 | 5 | 6 | 4 | 5 | 6 | 6 | 4 | 6 | 7 | 4 | 6 | 7 | 6 | 7 | 4 | 4 | 3 | 7 | 7 | 7 |
| ACOG-2021, FGR | | | | | | | | | | | | | | | | | | | | | | | |
| Rev-1 | 7 | 6 | 5 | 5 | 5 | 4 | 7 | 3 | 1 | 4 | 2 | 5 | 0 | 5 | 6 | 6 | 6 | 2 | 0 | 1 | 2 | 1 | 0 |
| Rev-2 | 6 | 6 | 6 | 6 | 4 | 5 | 6 | 4 | 2 | 3 | 3 | 4 | 1 | 6 | 6 | 5 | 7 | 1 | 1 | 0 | 1 | 1 | 1 |
| Rev-3 | 7 | 7 | 5 | 6 | 4 | 4 | 7 | 4 | 1 | 4 | 2 | 6 | 0 | 6 | 5 | 5 | 7 | 0 | 1 | 1 | 2 | 0 | 1 |
| Rev-4 | 6 | 6 | 6 | 5 | 5 | 5 | 7 | 2 | 2 | 5 | 3 | 6 | 1 | 5 | 6 | 5 | 7 | 1 | 1 | 2 | 1 | 1 | 0 |
| SOGC-2023, FGR | | | | | | | | | | | | | | | | | | | | | | | |
| Rev-1 | 7 | 7 | 5 | 5 | 3 | 5 | 5 | 7 | 5 | 7 | 5 | 6 | 5 | 6 | 7 | 7 | 7 | 2 | 6 | 5 | 5 | 6 | 7 |
| Rev-2 | 7 | 6 | 5 | 4 | 4 | 5 | 6 | 7 | 4 | 7 | 6 | 5 | 4 | 7 | 7 | 7 | 7 | 1 | 6 | 4 | 6 | 5 | 7 |
| Rev-3 | 7 | 6 | 6 | 6 | 6 | 5 | 3 | 2 | 5 | 2 | 4 | 5 | 2 | 6 | 6 | 6 | 5 | 2 | 1 | 0 | 1 | 3 | 7 |
| Rev-4 | 7 | 6 | 6 | 7 | 5 | 6 | 4 | 3 | 6 | 3 | 5 | 4 | 1 | 5 | 7 | 5 | 4 | 3 | 1 | 1 | 0 | 2 | 7 |
| RCOG-2024, FGR | | | | | | | | | | | | | | | | | | | | | | | |
| Rev-1 | 7 | 7 | 7 | 6 | 4 | 6 | 7 | 7 | 7 | 6 | 5 | 7 | 4 | 7 | 7 | 7 | 6 | 3 | 6 | 5 | 6 | 7 | 7 |
| Rev-2 | 7 | 6 | 6 | 6 | 3 | 5 | 7 | 7 | 6 | 7 | 4 | 7 | 3 | 7 | 7 | 7 | 5 | 2 | 5 | 6 | 5 | 6 | 7 |
| Rev-3 | 7 | 6 | 6 | 6 | 6 | 6 | 6 | 5 | 6 | 5 | 6 | 6 | 5 | 7 | 6 | 5 | 7 | 6 | 3 | 5 | 7 | 7 | 7 |
| Rev-4 | 7 | 7 | 7 | 6 | 5 | 6 | 5 | 6 | 6 | 4 | 6 | 7 | 4 | 7 | 6 | 6 | 7 | 5 | 4 | 4 | 6 | 7 | 7 |
| ACOG-2022, Advanced maternal age | | | | | | | | | | | | | | | | | | | | | | | |
| Rev-1 | 7 | 7 | 6 | 5 | 5 | 6 | 7 | 7 | 7 | 7 | 6 | 7 | 5 | 7 | 7 | 6 | 7 | 4 | 6 | 6 | 4 | 7 | 7 |
| Rev-2 | 7 | 7 | 5 | 5 | 6 | 7 | 7 | 7 | 7 | 7 | 7 | 7 | 4 | 7 | 7 | 5 | 7 | 5 | 6 | 7 | 5 | 6 | 7 |
| Rev-3 | 7 | 6 | 6 | 6 | 5 | 4 | 7 | 6 | 1 | 2 | 4 | 5 | 1 | 6 | 6 | 5 | 6 | 1 | 2 | 0 | 2 | 5 | 7 |
| Rev-4 | 7 | 6 | 7 | 6 | 4 | 5 | 7 | 6 | 2 | 1 | 5 | 4 | 0 | 7 | 5 | 6 | 7 | 0 | 3 | 1 | 1 | 6 | 7 |
| CMA-2024, Advanced maternal age | | | | | | | | | | | | | | | | | | | | | | | |
| Rev-1 | 7 | 6 | 3 | 5 | 1 | 3 | 4 | 1 | 7 | 3 | 5 | 6 | 4 | 0 | 7 | 6 | 7 | 1 | 3 | 3 | 1 | 4 | 7 |
| Rev-2 | 7 | 6 | 4 | 4 | 0 | 3 | 5 | 0 | 7 | 4 | 4 | 5 | 5 | 0 | 7 | 7 | 7 | 0 | 2 | 3 | 0 | 5 | 7 |
| Rev-3 | 7 | 6 | 6 | 7 | 5 | 4 | 7 | 6 | 4 | 7 | 6 | 5 | 1 | 1 | 7 | 6 | 6 | 4 | 3 | 1 | 5 | 0 | 7 |
| Rev-4 | 7 | 6 | 6 | 7 | 4 | 5 | 7 | 5 | 5 | 7 | 6 | 4 | 0 | 2 | 6 | 7 | 6 | 2 | 2 | 0 | 4 | 1 | 7 |
| FRENCH-2016, Pregnancy Loss | | | | | | | | | | | | | | | | | | | | | | | |
| Rev-1 | 6 | 5 | 4 | 4 | 3 | 5 | 2 | 6 | 5 | 3 | 4 | 5 | 3 | 0 | 6 | 6 | 4 | 1 | 2 | 4 | 4 | 0 | 0 |
| Rev-2 | 5 | 6 | 3 | 4 | 2 | 5 | 1 | 6 | 4 | 3 | 5 | 6 | 2 | 0 | 7 | 6 | 3 | 0 | 1 | 5 | 4 | 1 | 0 |
| Rev-3 | 7 | 6 | 7 | 5 | 5 | 6 | 3 | 4 | 2 | 4 | 5 | 6 | 3 | 6 | 7 | 6 | 6 | 2 | 3 | 4 | 0 | 0 | 7 |
| Rev-4 | 7 | 7 | 6 | 6 | 6 | 5 | 2 | 5 | 3 | 5 | 6 | 5 | 1 | 7 | 7 | 6 | 5 | 3 | 2 | 2 | 1 | 0 | 7 |
| QUEENSLAND-2022, Pregnancy Loss | | | | | | | | | | | | | | | | | | | | | | | |
| Rev-1 | 6 | 5 | 5 | 6 | 2 | 5 | 3 | 3 | 4 | 2 | 6 | 7 | 5 | 3 | 6 | 7 | 7 | 2 | 6 | 4 | 7 | 5 | 7 |
| Rev-2 | 5 | 6 | 4 | 6 | 1 | 4 | 2 | 2 | 5 | 1 | 7 | 7 | 5 | 2 | 6 | 7 | 7 | 1 | 5 | 5 | 7 | 4 | 7 |
| Rev-3 | 7 | 6 | 7 | 7 | 5 | 6 | 5 | 6 | 6 | 5 | 7 | 5 | 7 | 5 | 7 | 4 | 4 | 2 | 6 | 3 | 3 | 7 | 7 |
| Rev-4 | 7 | 7 | 7 | 6 | 6 | 7 | 4 | 6 | 6 | 4 | 6 | 4 | 7 | 6 | 7 | 5 | 6 | 1 | 5 | 2 | 2 | 6 | 7 |
| CMA-2022, Pregnancy Loss | | | | | | | | | | | | | | | | | | | | | | | |
| Rev-1 | 7 | 6 | 4 | 4 | 1 | 4 | 4 | 2 | 2 | 1 | 4 | 4 | 1 | 0 | 5 | 6 | 7 | 1 | 1 | 2 | 1 | 3 | 7 |
| Rev-2 | 6 | 7 | 3 | 4 | 2 | 3 | 4 | 1 | 2 | 0 | 5 | 4 | 0 | 0 | 6 | 7 | 7 | 2 | 0 | 2 | 2 | 3 | 6 |
| Rev-3 | 7 | 7 | 6 | 6 | 6 | 2 | 5 | 6 | 2 | 3 | 6 | 7 | 3 | 4 | 4 | 5 | 3 | 2 | 0 | 2 | 1 | 0 | 7 |
| Rev-4 | 7 | 6 | 7 | 6 | 5 | 1 | 6 | 5 | 1 | 4 | 6 | 7 | 2 | 5 | 5 | 4 | 4 | 1 | 1 | 1 | 2 | 0 | 7 |
| RCOG-2023, Pregnancy Loss | | | | | | | | | | | | | | | | | | | | | | | |
| Rev-1 | 7 | 6 | 6 | 6 | 4 | 6 | 7 | 7 | 7 | 5 | 6 | 7 | 5 | 7 | 7 | 7 | 7 | 3 | 6 | 5 | 5 | 7 | 7 |
| Rev-2 | 7 | 7 | 6 | 5 | 3 | 6 | 7 | 7 | 7 | 4 | 5 | 7 | 6 | 7 | 7 | 7 | 7 | 2 | 5 | 6 | 5 | 7 | 7 |
| Rev-3 | 7 | 7 | 7 | 6 | 7 | 7 | 6 | 7 | 6 | 5 | 5 | 7 | 5 | 7 | 6 | 6 | 6 | 6 | 3 | 5 | 6 | 6 | 7 |
| Rev-4 | 7 | 7 | 7 | 6 | 7 | 7 | 6 | 6 | 5 | 6 | 6 | 6 | 4 | 7 | 5 | 7 | 5 | 5 | 4 | 4 | 5 | 6 | 7 |
| ESHRE-2023, Pregnancy Loss | | | | | | | | | | | | | | | | | | | | | | | |
| Rev-1 | 7 | 7 | 7 | 7 | 6 | 7 | 7 | 7 | 7 | 7 | 6 | 7 | 7 | 7 | 7 | 7 | 7 | 6 | 7 | 7 | 6 | 7 | 7 |
| Rev-2 | 7 | 7 | 7 | 7 | 7 | 7 | 7 | 7 | 7 | 7 | 6 | 7 | 7 | 7 | 7 | 7 | 7 | 5 | 7 | 7 | 5 | 6 | 7 |
| Rev-3 | 7 | 7 | 7 | 6 | 6 | 6 | 6 | 6 | 5 | 4 | 6 | 7 | 5 | 6 | 7 | 6 | 6 | 5 | 6 | 4 | 6 | 6 | 7 |
| Rev-4 | 7 | 7 | 6 | 6 | 5 | 6 | 7 | 7 | 4 | 5 | 5 | 6 | 3 | 7 | 6 | 5 | 7 | 4 | 5 | 3 | 6 | 6 | 7 |
| SOGC-2018, Stillbirth | | | | | | | | | | | | | | | | | | | | | | | |
| Rev-1 | 7 | 7 | 7 | 6 | 6 | 7 | 6 | 7 | 7 | 6 | 6 | 6 | 5 | 6 | 7 | 6 | 7 | 3 | 5 | 4 | 7 | 7 | 7 |
| Rev-2 | 7 | 6 | 7 | 5 | 6 | 8 | 6 | 7 | 7 | 7 | 6 | 6 | 4 | 6 | 7 | 7 | 7 | 2 | 6 | 3 | 6 | 7 | 7 |
| Rev-3 | 7 | 6 | 7 | 6 | 6 | 6 | 7 | 7 | 6 | 4 | 5 | 6 | 5 | 7 | 6 | 5 | 7 | 4 | 3 | 5 | 6 | 6 | 6 |
| Rev-4 | 7 | 7 | 6 | 6 | 5 | 6 | 7 | 6 | 5 | 5 | 6 | 6 | 4 | 7 | 7 | 4 | 7 | 3 | 5 | 4 | 5 | 6 | 6 |
| FCOG-2024, Stillbirth | | | | | | | | | | | | | | | | | | | | | | | |
| Rev-1 | 7 | 5 | 5 | 7 | 2 | 7 | 6 | 4 | 7 | 7 | 5 | 6 | 6 | 4 | 7 | 5 | 6 | 3 | 5 | 6 | 5 | 5 | 7 |
| Rev-2 | 7 | 6 | 4 | 7 | 2 | 7 | 6 | 4 | 7 | 7 | 4 | 6 | 6 | 4 | 7 | 4 | 6 | 2 | 5 | 6 | 6 | 5 | 7 |
| Rev-3 | 7 | 7 | 6 | 7 | 6 | 6 | 7 | 6 | 6 | 7 | 6 | 7 | 2 | 1 | 5 | 4 | 6 | 2 | 5 | 6 | 5 | 7 | 7 |
| Rev-4 | 7 | 6 | 7 | 7 | 6 | 5 | 7 | 7 | 5 | 7 | 6 | 7 | 1 | 2 | 4 | 5 | 5 | 1 | 4 | 6 | 4 | 7 | 7 |
| ACOG-2018, Pre-eclampsia | | | | | | | | | | | | | | | | | | | | | | | |
| Rev-1 | 7 | 7 | 6 | 3 | 2 | 6 | 1 | 1 | 6 | 2 | 2 | 6 | 2 | 0 | 7 | 7 | 7 | 1 | 3 | 3 | 2 | 0 | 0 |
| Rev-2 | 6 | 6 | 6 | 2 | 3 | 3 | 0 | 2 | 6 | 2 | 1 | 3 | 1 | 1 | 3 | 7 | 4 | 0 | 2 | 2 | 1 | 0 | 0 |
| Rev-3 | 7 | 6 | 6 | 6 | 5 | 5 | 2 | 4 | 3 | 5 | 7 | 7 | 5 | 7 | 6 | 6 | 7 | 4 | 5 | 2 | 6 | 0 | 0 |
| Rev-4 | 7 | 6 | 6 | 5 | 6 | 5 | 1 | 5 | 4 | 5 | 6 | 7 | 5 | 7 | 6 | 7 | 7 | 5 | 4 | 1 | 5 | 0 | 0 |
| NEW ZEALAND-2018, Hypertension and Pre-eclampsia | | | | | | | | | | | | | | | | | | | | | | | |
| Rev-1 | 7 | 7 | 7 | 6 | 5 | 7 | 7 | 7 | 7 | 7 | 7 | 7 | 6 | 6 | 7 | 7 | 7 | 7 | 6 | 7 | 7 | 7 | 7 |
| Rev-2 | 7 | 7 | 7 | 7 | 6 | 7 | 7 | 7 | 7 | 7 | 7 | 7 | 7 | 6 | 7 | 7 | 7 | 7 | 5 | 7 | 7 | 7 | 7 |
| Rev-3 | 7 | 7 | 6 | 6 | 4 | 7 | 6 | 7 | 6 | 6 | 7 | 6 | 6 | 5 | 6 | 7 | 7 | 7 | 6 | 6 | 6 | 7 | 7 |
| Rev-4 | 7 | 6 | 7 | 6 | 6 | 7 | 7 | 6 | 6 | 7 | 7 | 6 | 5 | 6 | 7 | 7 | 6 | 7 | 6 | 6 | 5 | 7 | 7 |
| FIGO-2019, Pre-eclampsia | | | | | | | | | | | | | | | | | | | | | | | |
| Rev-1 | 7 | 6 | 6 | 5 | 4 | 7 | 4 | 6 | 7 | 5 | 6 | 6 | 5 | 4 | 6 | 6 | 7 | 6 | 5 | 6 | 5 | 7 | 7 |
| Rev-2 | 7 | 7 | 7 | 6 | 3 | 7 | 3 | 6 | 7 | 4 | 6 | 5 | 5 | 3 | 7 | 6 | 7 | 5 | 4 | 7 | 4 | 7 | 7 |
| Rev-3 | 7 | 6 | 7 | 5 | 4 | 6 | 4 | 5 | 7 | 3 | 5 | 4 | 6 | 3 | 6 | 5 | 6 | 5 | 6 | 6 | 3 | 7 | 7 |
| Rev-4 | 7 | 6 | 6 | 4 | 2 | 7 | 5 | 6 | 7 | 4 | 6 | 5 | 5 | 2 | 6 | 6 | 7 | 5 | 4 | 6 | 4 | 7 | 7 |
| ACOG-2020, Hypertension and Pre-eclampsia | | | | | | | | | | | | | | | | | | | | | | | |
| Rev-1 | 7 | 6 | 6 | 3 | 2 | 6 | 5 | 6 | 6 | 3 | 5 | 5 | 2 | 5 | 7 | 6 | 7 | 2 | 6 | 3 | 3 | 7 | 7 |
| Rev-2 | 7 | 7 | 7 | 1 | 2 | 5 | 4 | 7 | 6 | 2 | 4 | 4 | 1 | 6 | 7 | 7 | 7 | 1 | 5 | 2 | 2 | 6 | 6 |
| Rev-3 | 7 | 7 | 6 | 2 | 1 | 5 | 3 | 6 | 6 | 3 | 3 | 5 | 0 | 6 | 7 | 6 | 6 | 1 | 6 | 1 | 0 | 7 | 7 |
| Rev-4 | 7 | 7 | 6 | 1 | 2 | 6 | 4 | 6 | 5 | 2 | 4 | 5 | 1 | 6 | 7 | 6 | 7 | 1 | 5 | 2 | 2 | 6 | 7 |
| CMA-2020, Hypertension | | | | | | | | | | | | | | | | | | | | | | | |
| Rev-1 | 6 | 4 | 4 | 4 | 3 | 3 | 2 | 2 | 5 | 3 | 4 | 4 | 2 | 1 | 6 | 6 | 6 | 1 | 1 | 2 | 1 | 3 | 7 |
| Rev-2 | 6 | 5 | 3 | 3 | 2 | 1 | 0 | 3 | 6 | 3 | 5 | 5 | 1 | 0 | 7 | 6 | 6 | 0 | 1 | 1 | 0 | 4 | 6 |
| Rev-3 | 7 | 6 | 2 | 4 | 1 | 3 | 1 | 2 | 5 | 2 | 4 | 4 | 2 | 1 | 6 | 5 | 6 | 1 | 2 | 2 | 1 | 2 | 7 |
| Rev-4 | 7 | 5 | 3 | 3 | 2 | 1 | 0 | 1 | 5 | 3 | 5 | 3 | 1 | 0 | 7 | 6 | 5 | 0 | 1 | 1 | 0 | 4 | 7 |
| IAPM-2021, Pre-eclampsia | | | | | | | | | | | | | | | | | | | | | | | |
| Rev-1 | 7 | 5 | 3 | 2 | 1 | 3 | 0 | 1 | 1 | 0 | 4 | 3 | 1 | 0 | 4 | 5 | 2 | 1 | 2 | 1 | 1 | 3 | 6 |
| Rev-2 | 6 | 6 | 2 | 1 | 0 | 3 | 0 | 2 | 2 | 1 | 3 | 2 | 0 | 0 | 5 | 6 | 2 | 0 | 3 | 1 | 0 | 4 | 6 |
| Rev-3 | 6 | 6 | 1 | 0 | 1 | 2 | 0 | 3 | 2 | 1 | 4 | 3 | 1 | 0 | 4 | 5 | 3 | 1 | 2 | 0 | 1 | 2 | 6 |
| Rev-4 | 6 | 6 | 2 | 1 | 0 | 1 | 1 | 2 | 2 | 1 | 2 | 4 | 1 | 0 | 5 | 5 | 2 | 0 | 1 | 1 | 0 | 1 | 6 |
| QUEENSLAND-2021, Hypertension | | | | | | | | | | | | | | | | | | | | | | | |
| Rev-1 | 7 | 7 | 7 | 6 | 5 | 7 | 5 | 6 | 6 | 6 | 7 | 7 | 5 | 6 | 7 | 7 | 7 | 4 | 5 | 6 | 6 | 6 | 7 |
| Rev-2 | 7 | 7 | 7 | 5 | 5 | 7 | 4 | 5 | 5 | 5 | 6 | 7 | 5 | 5 | 7 | 7 | 7 | 4 | 6 | 5 | 5 | 6 | 7 |
| Rev-3 | 7 | 7 | 6 | 5 | 6 | 7 | 3 | 6 | 6 | 5 | 5 | 7 | 6 | 5 | 7 | 6 | 6 | 3 | 5 | 6 | 5 | 6 | 7 |
| Rev-4 | 7 | 7 | 6 | 6 | 5 | 7 | 4 | 6 | 5 | 5 | 6 | 7 | 5 | 5 | 7 | 7 | 7 | 4 | 5 | 5 | 5 | 6 | 7 |
| USPSTF-2021, Pre-eclampsia | | | | | | | | | | | | | | | | | | | | | | | |
| Rev-1 | 7 | 6 | 7 | 7 | 5 | 7 | 5 | 5 | 7 | 5 | 6 | 7 | 5 | 7 | 7 | 7 | 7 | 4 | 7 | 6 | 7 | 7 | 7 |
| Rev-2 | 7 | 7 | 7 | 7 | 4 | 6 | 4 | 6 | 7 | 6 | 5 | 7 | 6 | 7 | 7 | 7 | 7 | 5 | 7 | 5 | 6 | 7 | 7 |
| Rev-3 | 7 | 7 | 6 | 6 | 5 | 6 | 5 | 5 | 7 | 5 | 4 | 6 | 5 | 7 | 6 | 7 | 7 | 4 | 7 | 4 | 6 | 7 | 7 |
| Rev-4 | 7 | 7 | 6 | 7 | 6 | 6 | 4 | 4 | 7 | 5 | 5 | 7 | 5 | 7 | 7 | 6 | 7 | 4 | 7 | 5 | 6 | 7 | 7 |
| WHO-2021, Pre-eclampsia | | | | | | | | | | | | | | | | | | | | | | | |
| Rev-1 | 7 | 7 | 7 | 7 | 6 | 7 | 5 | 7 | 7 | 7 | 7 | 7 | 7 | 6 | 7 | 6 | 7 | 5 | 5 | 7 | 5 | 7 | 7 |
| Rev-2 | 7 | 7 | 7 | 7 | 7 | 7 | 6 | 6 | 7 | 7 | 7 | 7 | 7 | 5 | 6 | 7 | 7 | 4 | 3 | 6 | 4 | 7 | 7 |
| Rev-3 | 7 | 7 | 7 | 6 | 6 | 6 | 6 | 5 | 7 | 6 | 6 | 6 | 7 | 4 | 6 | 6 | 7 | 5 | 5 | 5 | 2 | 7 | 7 |
| Rev-4 | 7 | 7 | 6 | 5 | 6 | 7 | 6 | 6 | 7 | 7 | 7 | 7 | 6 | 5 | 6 | 7 | 7 | 4 | 4 | 6 | 4 | 7 | 7 |
| SOGC-2022, Hypertension | | | | | | | | | | | | | | | | | | | | | | | |
| Rev-1 | 7 | 6 | 7 | 5 | 5 | 7 | 3 | 4 | 5 | 4 | 6 | 5 | 3 | 6 | 7 | 7 | 6 | 4 | 4 | 4 | 3 | 5 | 5 |
| Rev-2 | 7 | 7 | 7 | 6 | 6 | 4 | 2 | 5 | 6 | 6 | 6 | 3 | 1 | 7 | 7 | 7 | 5 | 3 | 3 | 2 | 1 | 6 | 6 |
| Rev-3 | 7 | 6 | 7 | 5 | 6 | 5 | 1 | 4 | 5 | 4 | 5 | 4 | 3 | 6 | 7 | 6 | 5 | 2 | 4 | 3 | 3 | 5 | 6 |
| Rev-4 | 7 | 7 | 6 | 6 | 6 | 4 | 2 | 3 | 5 | 5 | 5 | 3 | 1 | 5 | 7 | 7 | 5 | 3 | 5 | 2 | 1 | 5 | 6 |
| SOMANZ-2023, Hypertension | | | | | | | | | | | | | | | | | | | | | | | |
| Rev-1 | 7 | 7 | 7 | 7 | 6 | 7 | 7 | 7 | 7 | 5 | 7 | 7 | 7 | 7 | 7 | 7 | 7 | 6 | 7 | 6 | 5 | 7 | 7 |
| Rev-2 | 7 | 7 | 7 | 7 | 5 | 7 | 7 | 7 | 7 | 5 | 7 | 7 | 7 | 7 | 7 | 6 | 7 | 5 | 7 | 5 | 5 | 7 | 7 |
| Rev-3 | 7 | 6 | 7 | 7 | 6 | 7 | 6 | 7 | 7 | 6 | 7 | 6 | 6 | 7 | 7 | 7 | 6 | 6 | 7 | 5 | 6 | 7 | 7 |
| Rev-4 | 7 | 7 | 7 | 6 | 6 | 7 | 7 | 6 | 7 | 5 | 7 | 6 | 7 | 7 | 7 | 6 | 7 | 6 | 7 | 5 | 5 | 7 | 7 |
| USPSTF-2023, Hypertension | | | | | | | | | | | | | | | | | | | | | | | |
| Rev-1 | 7 | 7 | 7 | 7 | 4 | 7 | 5 | 7 | 7 | 6 | 7 | 7 | 6 | 7 | 7 | 7 | 7 | 6 | 7 | 6 | 5 | 7 | 7 |
| Rev-2 | 7 | 7 | 7 | 7 | 5 | 7 | 4 | 7 | 7 | 5 | 7 | 7 | 5 | 7 | 7 | 7 | 7 | 5 | 7 | 5 | 6 | 7 | 7 |
| Rev-3 | 7 | 7 | 7 | 7 | 5 | 7 | 4 | 7 | 6 | 5 | 7 | 7 | 4 | 7 | 6 | 7 | 7 | 6 | 7 | 6 | 4 | 7 | 7 |
| Rev-4 | 7 | 6 | 6 | 7 | 5 | 7 | 4 | 7 | 7 | 5 | 7 | 7 | 5 | 7 | 7 | 7 | 7 | 6 | 7 | 5 | 5 | 7 | 7 |
| NICE-2023, Hypertension | | | | | | | | | | | | | | | | | | | | | | | |
| Rev-1 | 7 | 7 | 7 | 6 | 7 | 7 | 6 | 7 | 7 | 6 | 7 | 6 | 7 | 7 | 7 | 7 | 7 | 7 | 6 | 6 | 6 | 6 | 7 |
| Rev-2 | 7 | 7 | 7 | 7 | 7 | 7 | 5 | 7 | 7 | 6 | 7 | 5 | 7 | 7 | 7 | 7 | 7 | 7 | 6 | 6 | 5 | 7 | 7 |
| Rev-3 | 7 | 7 | 6 | 6 | 7 | 7 | 6 | 7 | 6 | 5 | 7 | 6 | 7 | 6 | 7 | 7 | 6 | 7 | 5 | 5 | 5 | 7 | 7 |
| Rev-4 | 7 | 7 | 7 | 6 | 7 | 6 | 5 | 7 | 6 | 6 | 7 | 6 | 7 | 7 | 6 | 7 | 7 | 7 | 5 | 6 | 6 | 7 | 7 |
| AOM-2023, Hypertension | | | | | | | | | | | | | | | | | | | | | | | |
| Rev-1 | 7 | 7 | 6 | 7 | 5 | 7 | 7 | 6 | 7 | 7 | 6 | 5 | 5 | 7 | 7 | 7 | 7 | 4 | 5 | 5 | 4 | 5 | 7 |
| Rev-2 | 7 | 6 | 7 | 7 | 6 | 7 | 7 | 7 | 7 | 7 | 5 | 3 | 4 | 6 | 6 | 7 | 7 | 5 | 6 | 6 | 3 | 6 | 7 |
| Rev-3 | 7 | 7 | 6 | 7 | 5 | 7 | 7 | 6 | 6 | 5 | 5 | 4 | 5 | 6 | 5 | 7 | 7 | 4 | 5 | 5 | 2 | 6 | 7 |
| Rev-4 | 7 | 7 | 6 | 7 | 6 | 7 | 7 | 6 | 5 | 6 | 5 | 3 | 5 | 6 | 6 | 7 | 7 | 3 | 5 | 6 | 3 | 6 | 7 |
| RANZCOG-2024, Pre-eclampsia | | | | | | | | | | | | | | | | | | | | | | | |
| Rev-1 | 7 | 7 | 7 | 7 | 7 | 7 | 7 | 7 | 7 | 6 | 7 | 7 | 6 | 7 | 7 | 7 | 7 | 6 | 7 | 7 | 6 | 7 | 7 |
| Rev-2 | 7 | 7 | 7 | 7 | 7 | 7 | 7 | 7 | 7 | 7 | 7 | 7 | 5 | 7 | 7 | 7 | 7 | 6 | 7 | 7 | 6 | 7 | 7 |
| Rev-3 | 7 | 7 | 6 | 7 | 7 | 7 | 6 | 7 | 7 | 6 | 6 | 7 | 5 | 6 | 7 | 7 | 7 | 5 | 6 | 7 | 5 | 7 | 7 |
| Rev-4 | 7 | 7 | 7 | 7 | 7 | 7 | 6 | 6 | 7 | 6 | 7 | 7 | 6 | 7 | 7 | 7 | 7 | 6 | 7 | 7 | 5 | 7 | 7 |

AGREE II, Appraisal of Guidelines for Research and Evaluation II; ACOG, American College of Obstetricians and Gynecologists; CMA, Chinese Medical Association; FCOG, French College of Gynaecologists and Obstetricians; SMFM, Society for Maternal-Fetal Medicine; SOGC, Society of Obstetricians and Gynecologists of Canada; RCOG, Royal College of Obstetricians and Gynaecologists; ESHRE, European Society of Human Reproduction and Embryology; SOGC, Society of Obstetricians and Gynecologists of Canada; FIGO, International Federation of Gynecology and Obstetrics; IAPM, International Academy of Perinatal Medicine; USPSTF, US Preventive Services Task Force; WHO, World Health Organization; SOMANZ, Society of Obstetric Medicine Medicine and New Zealand; NICE, National Institute for Health and Care Excellence; AOM, Association of Ontario Midwives; RANZCOG, Royal Australian and New Zealand College of Obstetricians and Gynaecologists; APS, antiphospholipid syndrome; FGR, fetal growth restriction.

**Supplementary Table 6. Total scores percentage for each individual AGREE II domain items**

| **Document** | | **Domain1,%** | **Domain2,%** | **Domain3,%** | **Domain4,%** | **Domain5,%** | **Domain6,%** |
| --- | --- | --- | --- | --- | --- | --- | --- |
| APS | ACOG-2012 | 81.94 | 62.50 | 40.63 | 76.39 | 2.08 | 0.00 |
|  | CMA-2020 | 91.67 | 68.06 | 29.69 | 86.11 | 5.21 | 89.58 |
| FGR | FCOG-2015 | 81.94 | 69.44 | 70.31 | 86.11 | 30.21 | 70.83 |
|  | CMA-2019 | 90.28 | 70.83 | 76.04 | 88.89 | 43.75 | 8.33 |
|  | SMFM-2020 | 86.11 | 51.39 | 65.10 | 97.22 | 43.75 | 93.75 |
|  | ACOG-2021 | 84.72 | 63.89 | 44.27 | 81.94 | 1.04 | 0.00 |
|  | SOGC-2023 | 87.50 | 68.06 | 62.50 | 87.50 | 29.17 | 75.00 |
|  | RCOG-2024 | 94.44 | 73.61 | 81.77 | 88.89 | 64.58 | 97.92 |
| Advanced maternal age | ACOG-2022 | 91.67 | 72.22 | 71.88 | 86.11 | 38.54 | 91.67 |
|  | CMA-2024 | 81.94 | 50.00 | 52.60 | 93.06 | 18.75 | 62.50 |
| Pregnancy Loss | FRENCH-2016 | 79.17 | 61.11 | 46.88 | 79.17 | 22.92 | 14.58 |
|  | QUEENSLAND-2022 | 83.33 | 68.06 | 63.02 | 84.72 | 46.88 | 87.50 |
|  | CMA-2022 | 84.72 | 44.44 | 38.54 | 70.83 | 5.21 | 52.08 |
|  | RCOG-2023 | 95.83 | 80.56 | 84.90 | 90.28 | 61.46 | 95.83 |
|  | ESHRE-2023 | 98.61 | 88.89 | 86.98 | 93.06 | 76.04 | 93.75 |
| Stillbirth | SOGC-2018 | 95.83 | 84.72 | 82.81 | 90.28 | 57.29 | 61.11 |
|  | FCOG-2024 | 86.11 | 79.17 | 73.44 | 72.22 | 57.29 | 91.67 |
| Hypertension  or/and  Pre-eclampsia | ACOG-2018 | 88.89 | 54.17 | 43.75 | 86.11 | 31.25 | 0.00 |
|  | NEW ZEALAND-  2018 | 97.22 | 86.11 | 91.67 | 97.22 | 89.58 | 100 |
|  | FIGO-2019 | 93.06 | 66.67 | 66.15 | 87.50 | 67.71 | 100 |
|  | ACOG-2020 | 94.44 | 33.33 | 54.17 | 94.44 | 27.08 | 93.75 |
|  | CMA-2020 | 63.89 | 25.00 | 27.60 | 83.33 | 0.00 | 66.67 |
|  | IAPM-2021 | 61.11 | 4.17 | 7.81 | 50.00 | 0.00 | 54.17 |
|  | QUEENSLAND-2021 | 97.22 | 81.94 | 75.00 | 97.22 | 65.63 | 91.67 |
|  | USPSTF-2021 | 95.83 | 93.33 | 78.65 | 97.22 | 77.08 | 100 |
|  | WHO-2021 | 98.61 | 90.28 | 89.06 | 93.06 | 60.42 | 100 |
|  | SOGC-2022 | 95.83 | 73.61 | 52.60 | 88.89 | 32.29 | 75.00 |
|  | SOMANZ-2023 | 98.61 | 91.67 | 93.75 | 95.83 | 80.21 | 100 |
|  | USPSTF-2023 | 97.22 | 87.50 | 85.94 | 98.61 | 80.21 | 100 |
|  | NICE-2023 | 98.61 | 94.44 | 90.10 | 97.22 | 82.29 | 97.92 |
|  | AOM-2023 | 94.44 | 91.67 | 78.65 | 94.44 | 57.29 | 89.58 |
|  | RANZCOG-2024 | 98.61 | 100 | 92.71 | 100 | 87.50 | 100 |

AGREE II, Appraisal of Guidelines for Research and Evaluation II; ACOG, American College of Obstetricians and Gynecologists; CMA, Chinese Medical Association; FCOG, French College of Gynaecologists and Obstetricians; SMFM, Society for Maternal-Fetal Medicine; SOGC, Society of Obstetricians and Gynecologists of Canada; RCOG, Royal College of Obstetricians and Gynaecologists; ESHRE, European Society of Human Reproduction and Embryology; SOGC, Society of Obstetricians and Gynecologists of Canada; FIGO, International Federation of Gynecology and Obstetrics; IAPM, International Academy of Perinatal Medicine; USPSTF, US Preventive Services Task Force; WHO, World Health Organization; SOMANZ, Society of Obstetric Medicine Medicine and New Zealand; NICE, National Institute for Health and Care Excellence; AOM, Association of Ontario Midwives; RANZCOG, Royal Australian and New Zealand College of Obstetricians and Gynaecologists; APS, antiphospholipid syndrome; FGR, fetal growth restriction.
